# Collaborate and explain on-the-fly: knowledge-based reasoning and learning in *ad hoc* teamwork

**DOI:** 10.3389/frai.2026.1765191

**Published:** 2026-06-03

**Authors:** Hasra Dodampegama, Mohan Sridharan

**Affiliations:** Institute of Perception, Action and Behavior, School of Informatics, University of Edinburgh, Edinburgh, United Kingdom

**Keywords:** *ad hoc* teamwork, ecological rationality, explainable agency, interactive learning, knowledge representation, non-monotonic logical reasoning

## Abstract

This paper focuses on *ad hoc teamwork*, the problem of enabling an AI agent to collaborate with other agents without prior coordination. Methods considered state of the art for *ad hoc* teamwork formulate it primarily as a learning problem, using a large labeled dataset of different situations to model the action choices of other agents (or agent *types*) and determine the actions of the *ad hoc* agent. Such datasets are not readily available in practical domains, and these methods lack transparency and make it difficult to rapidly revise existing knowledge (or models) in response to changes in the domain, team composition, or agents' capabilities. Our architecture for *ad hoc* teamwork embeds the principles of refinement, ecological rationality, interactive learning, and explainable agency, leveraging the complementary strengths of knowledge-based and data-driven methods for reasoning and learning. Specifically, for any given goal, our architecture enables an *ad hoc* AI agent to determine its actions through non-monotonic logical reasoning with: (a) prior domain-specific commonsense knowledge; (b) models learned and revised rapidly to predict the behavior of other agents; and (c) anticipated abstract future goals based on generic knowledge of similar situations in a pretrained Large Language Model. In addition, the *ad hoc* agent processes natural language descriptions and observations of other agents' behavior, using a combination of a pretrained Large Language Model and decision-tree induction to incrementally acquire and revise knowledge in the form of objects, actions, and axioms that govern domain dynamics. Furthermore, the *ad hoc* agent generates relational descriptions as on-demand explanations of its decisions and beliefs, and those of other agents, in response to various types of questions. We ground and experimentally evaluate the capabilities of our architecture in *VirtualHome*, a realistic, physics-based 3D simulation environment. We demonstrate reliable, efficient, transparent, and scalable performance, providing a substantial improvement in performance compared with a purely knowledge-based baseline, and comparable or better performance than a purely data-driven baseline while using orders of magnitude fewer resources.

## 1 Introduction

**Figure 1:**
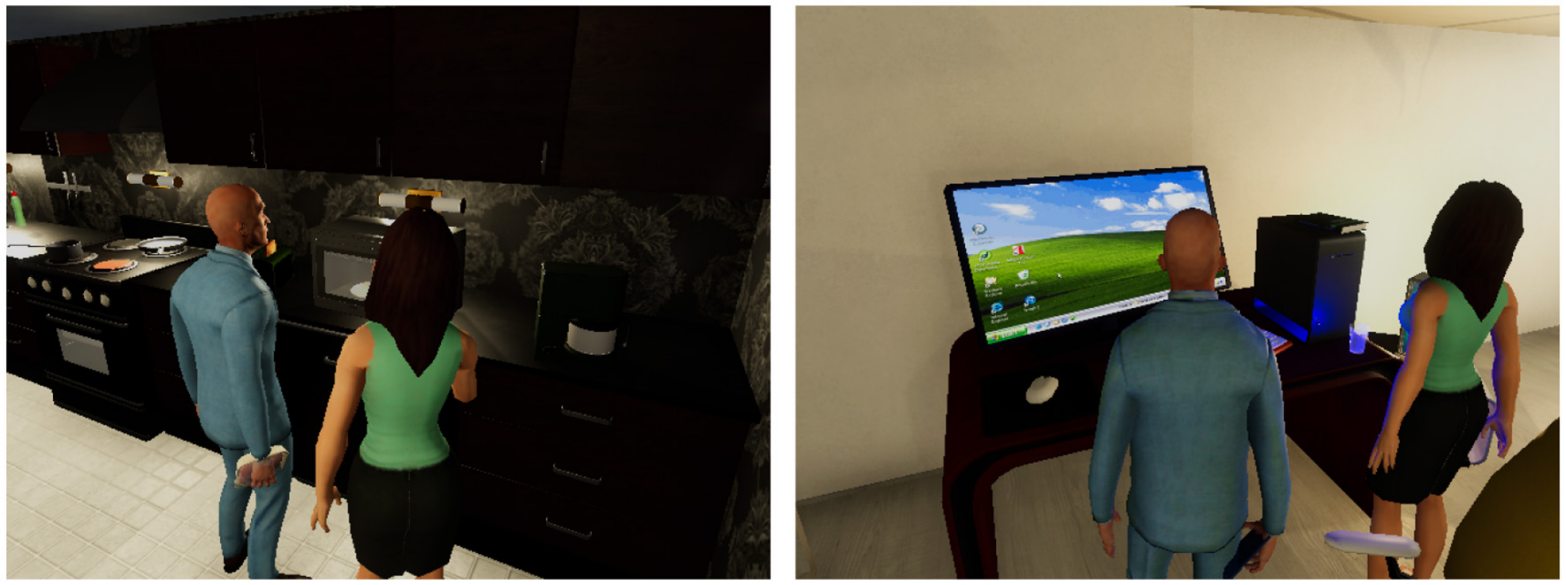
Screenshots from *VirtualHome* (Puig et al., 2018, 2020) showing a human (female in green top) and an assistive AI agent (male in blue shirt) collaborating.

Consider an AI agent performing household tasks in collaboration with other agents (AI, human) it has not worked with before. Figure 1 shows snapshots of this motivating scenario in which an AI agent (male, blue shirt) and a human (female, green top) are preparing breakfast and setting up a workstation. The agents have a limited view of the environment and do not communicate with each other, although each of them is aware of the domain state, including the location of teammates and the outcomes of actions, e.g., the change in the location of an object moved by a teammate. The AI agent has to reason with descriptions of prior knowledge and uncertainty that include qualitative statements (“eggs are usually in the fridge”) and quantitative measures of uncertainty (“I am 90% sure I saw the eggs on the kitchen table”). In addition, it has to operate under time exigencies, resource constraints, and changes in the domain, team composition, and the capabilities of the agents. Furthermore, the human may want to query and understand the decisions of the AI agent. These characteristics correspond to *ad hoc Teamwork* (AHT), requiring cooperation “on the fly” without prior coordination (Stone et al., 2010). The AHT problem arises in many practical applications such as disaster rescue, space exploration, and collaborative games, and poses challenges in knowledge representation, reasoning, and learning (Mirsky et al., 2022).

There has been considerable research in AHT, with initial methods using predefined protocols or *plays* designed to select the actions of the *ad hoc* agent in specific scenarios (Bowling and McCracken, 2005). Subsequent methods, especially those considered state of the art for AHT, include a key “data-driven” component in the form of probabilistic or deep network models that estimate the behavior of other agents (or agent *types*) and optimize the *ad hoc* agent's actions based on policies learned from a long history of prior interactions with similar agents (Barrett et al., 2013; Rahman et al., 2021; Ribeiro et al., 2023). While effective in specific scenarios, these methods have key limitations. First, they make it difficult to fully leverage the commonsense domain knowledge readily available in many practical domains. Secondly, these methods often require large amounts of data and computational resources for learning the models and policies that govern the AI agent's action choices, whereas practical AHT domains often impose strong resource constraints. Thirdly, it is challenging to deploy such models in AHT settings where previously unknown situations are common. Finally, the trained models often lack transparency, making it difficult to understand and audit the agent's decisions.

In a departure from existing work, our AHT architecture leverages the complementary strengths of knowledge-based and data-driven methods for reasoning and learning to jointly address the underlying challenges. It embeds fundamental principles such as refinement, ecological rationality, interactive learning, and explainable agency, which can be traced back to the early pioneers of AI (Sridharan, 2025), enabling each *ad hoc* agent in a team to:

1. Perform non-monotonic logical reasoning at two (formally-coupled) abstractions with prior commonsense domain knowledge, and models learned rapidly from limited examples to predict the behavior of each teammate, computing and executing a sequence of actions to collaborate with teammates to achieve the assigned goals;
2. Leverage a pre-trained Large Language Model (LLM) to anticipate future high-level tasks to be completed by the team, automatically prompting and adapting the LLM's output based on domain-specific knowledge, and assigning the current and anticipated tasks as joint goals to be achieved through non-monotonic logical reasoning;
3. Automatically construct on-demand relational descriptions as *explanations* of its decisions and beliefs, and those of other agents in the team, in response to different types of questions (e.g., causal, contrastive, and counterfactual); and
4. Incrementally acquire previously unknown knowledge in the form of new objects, actions, and axioms, and cumulatively revise existing knowledge, based on LLM-based processing of natural language descriptions of actions and outcomes, and decision tree induction applied to observations.

Our previous papers have described a subset of these capabilities, e.g., a proof of concept architecture for non-monotonic logical reasoning with prior commonsense knowledge and simple behavior prediction models (Dodampegama and Sridharan, 2023a,c), and an architecture that enables an agent to provide relational descriptions as explanations of its decisions (Dodampegama and Sridharan, 2024). This paper describes the entire architecture, emphasizing the benefits of leveraging the interplay between representation, reasoning, explanation generation, and learning; and discusses the qualitative and quantitative results of experimental evaluation with larger teams in more complex domains.

We ground and experimentally evaluate the capabilities of our architecture in the context of two or more agents (AI, human) collaborating to complete household tasks in *VirtualHome*, a realistic physics-based 3D simulation environment for multiagent collaboration (Puig et al., 2018). We use Answer Set Programming (ASP) (Gelfond and Kahl, 2014) for non-monotonic logical reasoning, and GPT4o mini (OpenAI and team, 2024) as the LLM. We show experimentally that our architecture leads to reliable, efficient, transparent, and scalable performance, providing a substantial improvement in performance compared with a purely knowledge-based baseline, and comparable or better performance than a purely data-driven baseline while using orders of magnitude fewer resources.

The remainder of the paper is organized as follows. We begin with a discussion on related work in Section 2, and describe our AHT architecture and its components in Section 3. Next, we describe the experimental setup, hypotheses, and the results of experimental evaluation in Section 4, with the conclusions summarized in Section 5.

## 2 Related work

Research in *ad hoc* Teamwork (AHT) has been carried out for at least two decades under different names (Mirsky et al., 2022). Initial methods for AHT relied on predefined protocols (or plays) designed for different scenarios, enabling an agent to choose (or plan) a sequence of protocols from the play library based on the current state (e.g., location of all agents and domain objects) (Bowling and McCracken, 2005). While these methods provide well-structured, transparent, and repeatable decisions, they lack flexibility in adapting to novel or dynamic environments. Subsequent work used probabilistic and sampling-based methods such as Upper Confidence bounds for Trees (UCT) to determine the *ad hoc* agent's actions (Barrett et al., 2013; Albrecht and Stone, 2017). This includes work that extended UCT by incorporating history information into nodes, reducing the number of distinct states represented in the search tree (Yourdshahi et al., 2018); and combined UCT with biased adaptive play (BAP) to form an online planning algorithm for AHT, with UCT estimating the joint utility function in each stage and BAP selecting the *ad hoc* agent's action based on that estimate (Wu et al., 2011). Other work has used the Bellman optimality equations to compute the *ad hoc* agent's actions by simulating interaction trajectories for certain known types of agents in the team (Albrecht and Ramamoorthy, 2013).

Over time, AHT has primarily been posed as a learning problem, incorporating different ideas to make this learning more tractable and to adapt to different situations. The key component of frameworks developed and considered state of the art for AHT has used probabilistic, reinforcement learning (RL), and/or deep neural network methods and a long history of prior interactions with similar agents or agent types, to predict the behavior of other agents and determine the *ad hoc* agent's action choices. For example, the PLASTIC algorithm used decision-tree models learned offline for each teammate type to probabilistically determine the *ad hoc* agent's actions, or used Fitted Q-Iteration with a set of learned policies for different types of teammates to determine the actions of the *ad hoc* agent (Barrett et al., 2017). Attention-based recurrent neural networks have been used, with one attention network for each (known) type of teammate, to extract temporal correlation between current and previously observed states and measure similarity between past and new teammates, enabling the *ad hoc* agent to adapt its actions based on its estimate of the type of agent(s) it is in interaction with (Chen et al., 2020). The PLASTIC-Policy under Adversarial Selection (PPAS) framework used a Deep Q-Network to learn a policy for different teams, collecting the transitions observed while interacting with a particular type of team in a separate KD-tree, and combining the output from the learned policies using a greedy weighted majority rule to determine the *ad hoc* agent's actions with non-stationary teammates (Santos et al., 2021). Another example is the use of model-based RL methods to determine the *ad hoc* agent's action choices, with a set of feed-forward neural networks trained using data of observed states and teammates actions to obtain a library of teammate behavior models, along with estimates of transition probabilities and a reward function (Ribeiro et al., 2023; Xu et al., 2025).

The learned models for types of teammates (and teams) have been used to simulate future interactions under different situations. For example, the learned models of different types of agents have been used to anticipate the effects of other agents' actions on the *ad hoc* agent's rewards in order to compute the policy governing the *ad hoc* agent's actions (Albrecht et al., 2015). The *ad hoc* agent's policy learned using proximal policy optimization has been conditioned on its belief about teammates, using sequential and hierarchical variational autoencoders to model changing behaviors of teammates by separating consistent behavior from short term changes (Zintgraf et al., 2021). Potential changes in team composition have also been handled by training Graph Neural Networks to consider a range of team sizes and teammate types, modeling teammates as nodes (in a graph) that can be added or removed, and using Long Short-term Memory networks for type inference (Rahman et al., 2021). More recent work has used a self-play strategy to learn a finite set of candidate teammate policies, using these policies to determine the cooperation policy for an *ad hoc* agent (Zand et al., 2022; Fang et al., 2024).

While the methods and frameworks summarized above offer promising strategies for modeling the behavior of teammates and selecting the actions of the *ad hoc* agent, they are resource-hungry, requiring substantial time, computation, and training examples to achieve the observed performance. These resources are not readily available in practical AHT domains, posing questions about the scalability and usability of these methods. Also, these methods struggle to leverage the commonsense knowledge available in many practical domains, and their internal mechanisms governing the decisions made are often opaque, limiting the ability to understand, justify, and audit the automated decisions made by the agents.

With Large Language Models (LLMs) and other foundation models (FMs) increasingly being considered state of the art for different problems in AI and robotics, they have been used for AHT as well. For example, an LLM-based hierarchical decision making system has been used to generate the *ad hoc* agent's policy and support zero-shot collaboration (Liu et al., 2024), and a framework has been developed to use memory retrieval and code-driven reasoning for AHT in the AvalonPlay benchmark (Shi et al., 2023). Physically realistic simulation environments such as Habitat (Savva et al., 2019) and VirtualHome (Puig et al., 2018) have been used to generate complex scenarios that serve both to train and evaluate the AHT frameworks based on LLMs and other data-driven methods. While frameworks based on such FMs have provided impressive experimental results in specific applications, there is increasing evidence to show that they can make arbitrary decisions in novel situations (Lu et al., 2024), do not plan, struggle to solve problems that require multi-step plans, and are more effective when used to generate abstract guidance that is validated before being used (Guan et al., 2023; Kambhampati et al., 2024).

There has been considerable research in the development and use of action languages and logics for multiagent domains. This includes action language A for an agent computing cooperative actions in multiagent domains (Son and Sakama, 2010), and recent work on action language mA* that introduces action types, epistemic planning, and dynamic awareness to model realistic interactions (Baral et al., 2022). Since agents operating in complex domains often need to revise their knowledge over time, many methods have been developed to support this ability using logics. For example, a system based on inductive logic programming has been developed to learn new knowledge in the form of an ASP program for practical applications (Law et al., 2018). Other approaches have used non-monotonic logical reasoning together with inductive learning and relational reinforcement learning to identify new rules for answer set programs (Sridharan et al., 2017). In this paper, we build on an existing action language for representing prior knowledge, and present a strategy that leverages an LLM and decision-tree induction to reliably and rapidly revise existing knowledge.

The increasing use of AI methods in different applications has led to considerable interest in providing transparency in the operation of such methods (Miller, 2019; Rudin et al., 2022), although this has been a well-researched area for decades (Reiter, 1987; Pearl, 2009). With such AI methods being used to interact with other agents and humans, multiple theories and approaches have been developed and adapted to define and use causal relationships to provide explanations (Halpern and Pearl, 2005), categorize methods in terms of their ability to extract causal relationships and provide explanation (Pearl and Mackenzie, 2018), and consider the recipient's prior knowledge while generating user-centric explanations (Ehsan et al., 2024; Finzel et al., 2021). Instead of assuming comprehensive prior knowledge or exploring black box models, we seek to design architectures that incorporate interpretable models (Rudin, 2019) and automatically support process-level explanations based on knowledge that is evolving over time. To achieve this objective, work in our group has designed architectures that embed the principle of *explainable agency* (Langley, 2019; Langley et al., 2017) and build on a theory for explanation generation (Sridharan and Meadows, 2019), enabling an agent to construct relational descriptions as explanations in response to different types of questions (Mota et al., 2021). The novelty is in the processes used to achieve the desired functional capabilities. In this paper, we extend these ideas and leverage the principle of ecological rationality (Gigerenzer, 2020) to provide explanations in the context of multiagent (*ad hoc*) collaboration.

Overall, our architecture poses AHT as a joint reasoning and learning problem. It embeds a set of fundamental principles that can be traced back to the early pioneers of AI (Sridharan, 2025), and builds on the complementary strengths of knowledge-based and data-driven methods. Some subset of the capabilities of this architecture have been described in our prior conference papers, e.g., a proof of concept architecture for non-monotonic logical reasoning with prior commonsense knowledge and simple behavior prediction models (Dodampegama and Sridharan, 2023a,c), an architecture that enables an *ad hoc* agent to provide relational descriptions as explanations of its decisions (Dodampegama and Sridharan, 2024), an architecture that demonstrates the ability to acquire domain knowledge in the form of some axioms (Dodampegama and Sridharan, 2023b), and an architecture that performs proof of concept exploration of scalability (Dodampegama and Sridharan, 2025b,a). Here we describe the entire architecture, explicitly exploring and highlighting the benefits of leveraging the interplay between the underlying reasoning, explanation generation, and learning problems. We also describe the results of thoroughly evaluating the reliability, efficiency, transparency, knowledge acquisition, and scalability of the architecture in complex, realistic household domains.

## 3 Methodology

**Figure 2:**
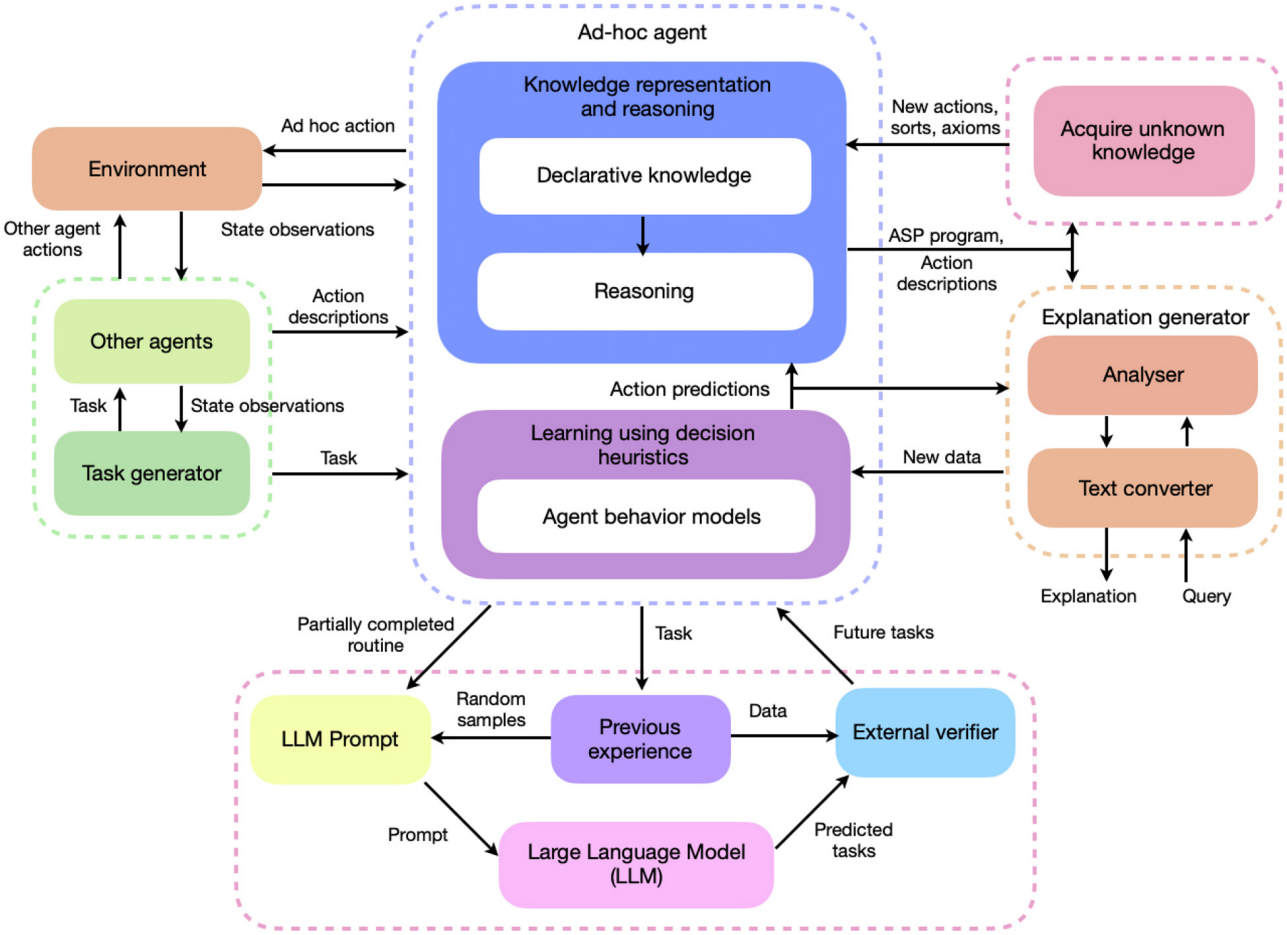
KAT embeds the principles of refinement, ecological rationality, interactive learning, and explainable agency to leverage the complementary strengths of knowledge-based and data-driven reasoning and learning.

**Figure 3:**
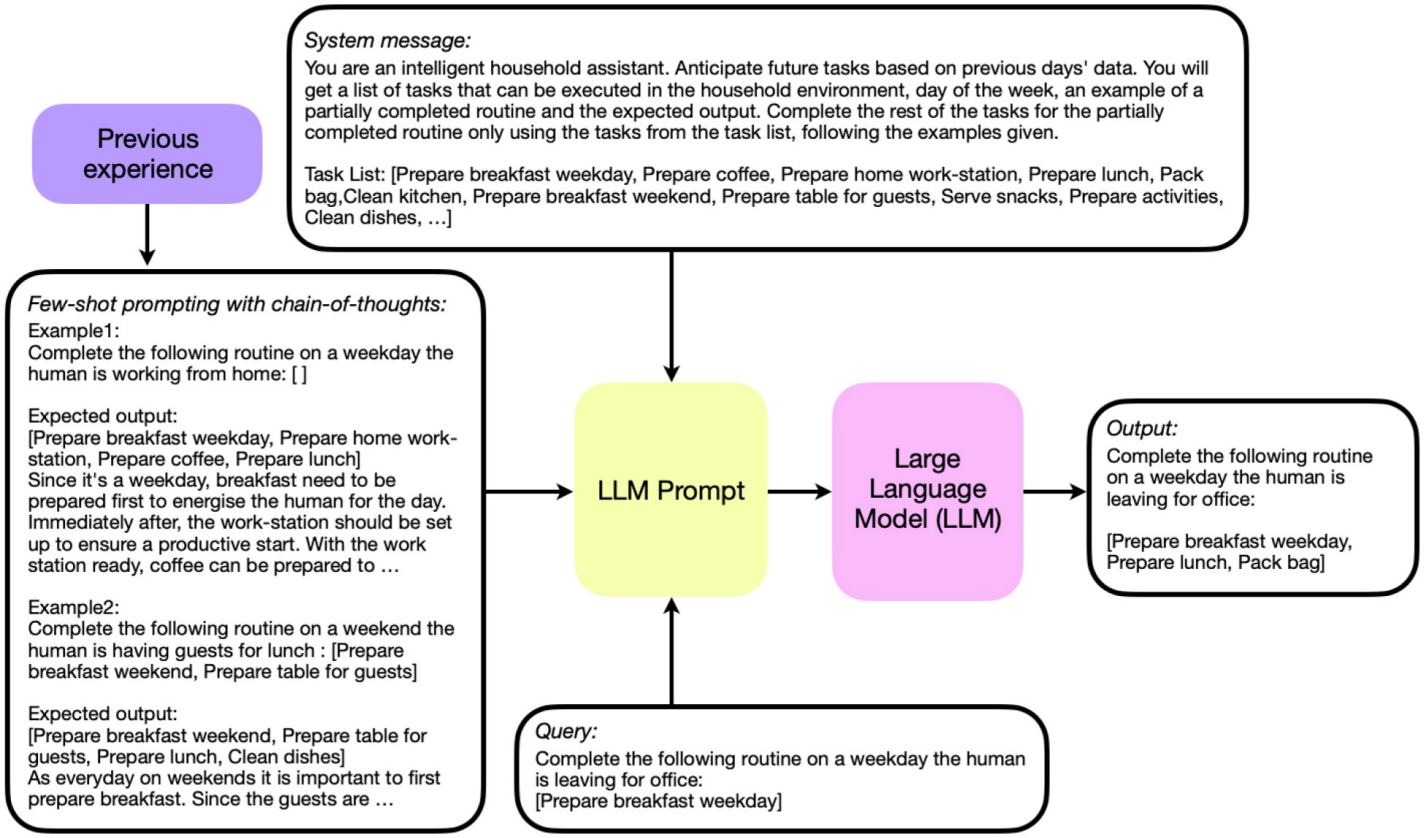
Example prompt sent to the LLM to anticipate the human's future tasks. Each prompt includes a list of candidate tasks, two partially completed example routines, and a partially completed routine for the LLM to complete.

Figure 2 outlines our architecture for Knowledge-guided AHT (KAT), which we describe in the context of agent (human, AI) collaborating to complete household tasks. To simulate a realistic (and evolving) daily routine, an external *task generator* produces a sequence of tasks for any given day (e.g., “make breakfast,” “set up workstation,” “prepare lunch”) and dispatches tasks one at a time to all agents. Each agent is unaware of the strategy driving the task generator and starts with no prior knowledge of the preferences, skills, and strategy of other agents, although it knows that teammates will collaborate toward achieving the common goal(s). In the baseline mode of operation, each agent receives information about the current state, and independently compute and execute actions to complete the task(s). The *ad hoc* agent determines its action by reasoning with domain knowledge (Section 3.1) and the predicted actions of other agents that are based on models learned and revised using runtime observations (Section 3.2). Moreover, the *ad hoc* agent uses a pretrained *LLM* to anticipate (high-level) future tasks in the environment, automatically prompting and adapting the LLM's output based on domain-specific knowledge, and jointly planning actions to achieve the current and anticipated tasks (Figure 3, Section 3.3). In addition, the human (agent) occasionally describes an agent's actions (e.g.,“Agent 1 cannot put the cake inside the microwave since the microwave's door is closed”) which are used by the *ad hoc* agent to learn previous unknown domain knowledge in the form of objects, actions, and axioms. The *ad hoc* agent also uses observations obtained during plan execution to learn and revise axioms based on decision tree induction (Section 3.5). Furthermore, the *ad hoc* agent provides relational descriptions as explanations of its decisions and beliefs, and those of other agents, in response to different types of questions (Section 3.4). We describe KAT's components for one *ad hoc* agent and a human using the example scenario given below. We will then consider multiple *ad hoc* agents when we experimentally explore the scalability of our architecture in Section 4.

** Example 1**. [**Human-AI agent collaboration scenario**]Consider an AI (*ad hoc*) agent and a human agent collaborating to complete daily household tasks such as preparing breakfast or setting up the home work-station; Figure 1 shows snapshots of such interactions in the VirtualHome domain. The agents can interact with the environment through actions that involve moving to places, picking up or placing objects, switching appliances on or off, and opening or closing appliances. Completing a task requires a sequence of such actions to be computed and executed by members of the team without any direct communication between them.^1^ The *ad hoc* agent assumes that its teammate will have access to the same information about domain state, predicts the actions that the teammate will execute over the next few steps, and computes its plan to complete the current task and prepare for the upcoming task(s). The *ad hoc* agent's prior commonsense knowledge includes relational descriptions of some attributes of the domain, objects, itself, and the human. It also includes axioms governing actions and changes, e.g., it is aware that it cannot hold more than two objects at a time and that it can only pick up objects that are next to it. We use this scenario because it allows us to thoroughly explore the reasoning, explanation generation, and learning problems observed in AHT domains.

### 3.1 Knowledge representation and reasoning

In KAT, any given domain's transition diagram is described using an extension of *action language* ${\mathrm{AL}}_{d}$ (Gelfond and Inclezan, 2013). Action languages are formal models of parts of natural language for describing transition diagrams of dynamic systems. The domain representation comprises a system description D, a collection of statements of ${\mathrm{AL}}_{d}$, and a history H. D has a sorted signature Σ with basic sorts, and domain attributes (statics, fluents) and actions described in terms of these sorts. Basic sorts in our example scenario include *location*, *object*, *appliance*, *ad*_*hoc*_*agent*, *human*, and *step* (for temporal reasoning), and are arranged hierarchically, e.g., *apple* is a sub-sort of *food*, a sub-sort of *graspable*, a sub-sort of *object*. Actions can be *agent*_*actions* or *exo*_*actions* (exogenous actions). The *agent*_*actions* such as *grab*(*ad*_*hoc*_*agent, object*), *switch*_*on*(*ad*_*hoc*_*agent, appliance*), *move*(*ad*_*hoc*_*agent, location*1, *location*2) are performed by the *ad hoc* agent. The *exo*_*actions* such as *exo*_*grab*(*other*_*agent, object*), *exo*_*switch*_*on*(*other*_*agent, appliance*) are performed by other agents, e.g., human or another AI agent. Statics are domain attributes whose values cannot change. Fluents are attributes whose values can change; they can be *inertial*, which obey inertia laws and are changed by the *ad hoc* agent's actions, e.g., *at*(*ad*_*hoc*_*agent, location*) is the *ad hoc* agent's location, or *defined*, which do not obey inertia laws and are not directly changed by the *ad hoc* agent's actions, e.g., *agent*_*at*(*other*_*agent, location*) is a teammate's location computed by (say) external sensors.

Given a specific Σ, the domain's dynamics are described using three types of axioms: *causal law, state constraint*, and *executablility condition* such as:


$$\begin{array}{ll} open\left(A,E\right) & \;\text{c}ause\text{s}\;opened\left(E\right) \end{array}$$ (1)

$$\begin{array}{ll} \neg at\left(A,L1\right) & \;\text{i}\text{f}\;at\left(A,L2\right),L1\neq L2 \end{array}$$ (2)

$$\begin{array}{ll} \text{i}mpossibl\text{e}\; & grab\left(A,O\right)\;\text{i}\text{f}\;on\left(O,E\right),\;\neg opened\left(E\right) \end{array}$$ (3)

where Statement 1(a), a causal law, implies that an agent (*A*) executing the *open*(*A, E*) action causes an appliance *E* to be opened; Statement 1(b), a state constraint, implies that an agent (*A*) cannot be in two places (*L*1, *L*2) at the same time; and Statement 1(c), an executability condition, prevents the agent (*A*) from trying to grab an object (*O*) from an appliance (*E*) that is not open.

The history (H) of a domain is a record of statements of the form *obs*(*fluent, boolean, step*), which represent observations, and of the form *hpd*(*action, step*), which represent executed actions, at specific steps. In KAT, H also includes default statements that are true in the initial state.

To reason with knowledge, our architecture uses a script to automatically construct program Π(D,H) by translating the system description D and history H to CR-Prolog (Balduccini and Gelfond, 2003), an extension to ASP that supports consistency restoring (CR) rules. Π(D,H) contains the ASP translation of statements from D and H, inertia axioms, reality check axioms, closed world assumptions for defined fluents and actions, and helper relations to reason over time steps, e.g., *holds*(*fluent, step*) and *occurs*(*action, step*) imply (respectively) that a fluent is true, and an action is part of a plan at a particular time step. It also contains helper axioms that define goals and guide planning and diagnosis. ASP is based on stable model semantics, and encodes *default negation* and *epistemic disjunction*, i.e., unlike “¬*a*” that states *a is believed to be false*, “*nota*” only implies *a is not believed to be true*, and unlike “*p* ∨¬*p*,” “*por* ¬*p*” is not tautologous. Each literal is true, false, or unknown, and the agent only believes that which it is forced to believe. ASP supports non-monotonic logical reasoning, i.e., the ability to revise previously held conclusions, which is essential for agents operating in practical domains with incomplete knowledge and noisy observations. The CR rules allow the agent to make assumptions under exceptional circumstances to recover from inconsistencies, e.g., if a book is not observed to be in the library (its default location) during plan execution, the agent reasons that the book was not initially in the library or was moved from there in a previous time step. All reasoning tasks, i.e., planning, diagnostics, and inference are then reduced to computing *answer sets* of Π subject to some criteria (e.g., minimize costs) and extracting the action sequence; we do so using the SPARC system (Balai et al., 2013).

Consider, for example, the scenario in which the goal of the team comprising a human and an *ad hoc* (assistive AI) agent is to prepare breakfast by preparing and bringing cereal, milk, and toast to the table. The *ad hoc* agent uses a learned model to predict the potential action of the human (as described in Section 3.2 below). Then, as described above, the agent translates all this information into an ASP program that it solves to compute an answer set that describes a potential world in which the desired goal is achieved over a sequence of time steps. This answer set contains literals such as:


$$\begin{array}{l} occurs\left(move\left(ad\text{\_}hoc\text{\_}agent,fridge\right),0\right),\\ occurs\left(open\left(ad\text{\_}hoc\text{\_}agent,fridge\right),1\right),\\ occurs\left(grab\left(ad\text{\_}hoc\text{\_}agent,milk\right),2\right),\\ \ldots ,holds\left(in\text{\_}hand\left(ad\text{\_}hoc\text{\_}agent,milk\right),3\right) \end{array}$$

in which the literals of the form *occurs(A, T)* indicate the action (A) to be executed by the agent at a specific time step (T), e.g., the agent is to move to the fridge and fetch milk from it, and the literals of the form *holds(F, T)* indicate the state of the world at specific time step, e.g., the agent is expected to be holding the milk carton at step 3. From this answer set, the agent extracts the literals that denote the plan to be executed by it, e.g., *move(ad_hoc_agent, fridge), open(ad_hoc_agent, fridge), grab(ad_hoc_agent, milk)*, and other actions (not shown here), which, in conjunction with the actions it expects the human to execute, will enable the team to achieve the desired goal. This action sequence is then executed in the simulated *VirtualHome* domain, with the corresponding observations and outcomes being added to H. Example SPARC programs for our example scenario, along with the scripts used to convert them, are in our code repository (Dodampegama and Sridharan, 2026).

Our example scenario is complex, with many objects, containers, and locations, e.g., there can be ≈10^25^ states with just one *ad hoc* agent and one human, making it computationally expensive to compute plans with multiple steps. To ensure scalability, we build on prior work in our group on a refinement-based architecture (Sridharan et al., 2019), which enables the *ad hoc* agent to represent and reason about its domain in the form of transition diagrams at two different resolutions. Specifically, a fine-resolution description ($D_{F}$) is defined as a *refinement* of a coarse-resolution description ($D_{C}$), with the agent now able to reason about aspects of the domain that were previously abstracted away. In our example scenario, the domain is organized into abstract regions in $D_{C}$, with each region being refined in $D_{F}$ into smaller regions that are components of the larger region, e.g., kitchen in $D_{C}$ comprises kitchen table, kitchen counter, refrigerator in $D_{F}$. In a similar manner, an object in $D_{C}$, e.g., a cup, can comprise multiple parts, e.g., handle and body, in $D_{F}$. The signature Σ_*F*_ of $D_{F}$ is created first by expanding Σ_*C*_ of $D_{C}$ to include the new sorts, actions, fluents, and statics. Next, the axioms of $D_{F}$ are defined by inheriting and adapting some actions from $D_{C}$ to the new (expanded) signature, and defining suitable *bridge axioms* that link the two descriptions. The axioms of $D_{L}$ for our example scenario include:


$$\begin{array}{l} move^{*}\left(A,L\right)\;\text{c}ause\text{s}\;at^{*}\left(A,L\right) \end{array}$$ (4)

$$\begin{array}{l} at\left(A,Rg\right)\;\text{i}\text{f}\;at^{*}\left(A,L\right),component\left(L,Rg\right) \end{array}$$ (5)

$$\begin{array}{ll} \text{i}mpossibl\text{e}\; & grab\left(A,O\right)\;\text{i}\text{f}\;at^{*}\left(A,L_{1}\right),at^{*}\left(O,L_{2}\right),L_{1}\neq L_{2} \end{array}$$ (6)

where *L* refers to grid-based locations in $D_{F}$ that are a *component of* a region-based location *Rg* in $D_{C}$, and superscript “*" refers to relations introduced in $D_{F}$. This coupling between descriptions provides clear separation of domain-dependent and domain-independent parts of the architecture, and provides conditions under which we can guarantee that any given transition in $D_{C}$ can be implemented as a sequence of transitions in $D_{F}$. In addition, it allows us to introduce (in $D_{F}$) observations and non-deterministic knowledge-producing actions, and to associate probabilities with action outcomes. Prior work in our group has demonstrated how $D_{F}$ can be automatically translated to probabilistic sequential decision-making formulations for planning.

Reasoning with the entire fine-resolution description can quickly become computationally intractable for complex domains. Our architecture addresses this problem by incorporating the related principle of *attention*. Specifically, the formal coupling between the two descriptions is leveraged to enable an *ad hoc* agent to automatically identify and *zoom to* the part of $D_{F}$ that is relevant to any given goal and abstract action under consideration. For example, an agent moving from an *office* to the *kitchen* to fetch a cup of hot tea can focus on just these two rooms and their components while ignoring the other rooms in the domain. Specifically, sorts and ground object instances in the signature Σ_*F*_ of $D_{F}$ are automatically restricted to those that are relevant to the abstract action (transition) to be implemented, and the axioms of $D_{F}$ are restricted to this reduced signature. This process substantially reduces the complexity of reasoning at the finer-granularity, leading to computationally efficient operation.

A common criticism of reasoning methods is that they need comprehensive domain knowledge, but architectures that embed key principles such as refinement have demonstrated the ability to reason with the available knowledge and revise it incrementally over time (Sridharan and Meadows, 2018). Also, most of the steps for encoding the available knowledge can be automated, and the effort involved in encoding prior knowledge is much less than that needed to train purely data-driven systems.

### 3.2 Agent behavior models

Reasoning with just prior domain knowledge that can be incomplete or inconsistent will lead to poor performance, particularly under AHT settings (see Section 4.2). Hence, KAT enables the *ad hoc* agent to also reason with models that predict the action choices of other agents and are learned (and revised) rapidly. This capability is achieved by embedding the principle of *Ecological Rationality* (ER) (Gigerenzer, 2020), which is based on Herb Simon's original definition of Bounded Rationality (Simon, 1956) and the algorithmic theory of decision heuristics (Gigerenzer and Gaissmaier, 2011). ER explores decision making “in the wild,” i.e., under open world uncertainty with the space of possibilities not fully known a priori, and characterizes behavior as a joint function of the agent's internal cognitive processes and the environment. It advocates the use of *adaptive satisficing* for making decisions in open worlds because in the absence of comprehensive knowledge, optimal decisions may be unknowable and not just hard to compute. Thus, decision heuristics (e.g., tallying, sequencing, fast, and frugal methods) are used to ignore part of the information, and to make decisions more quickly, frugally, and accurately than sophisticated methods with many more free parameters (Gigerenzer and Gaissmaier, 2011). In the current era of FMs, a large number (e.g., billions) of parameters are considered to be essential for building a single model or policy that generalizes across different situations, domains, and agent platforms. As discussed in Section 2, this design choice leads to multiple problems in practical domains. ER, on the other hand, focuses on building simpler models with limited free parameters, which can be learned and revised rapidly while using limited resources (e.g., computation, training examples). Such an approach has been shown to avoid overfitting and provide better performance than more sophisticated methods in practical applications while requiring much fewer resources (Gigerenzer, 2016), but decision heuristics and their impressive performance do not receive the attention that they deserve in the AI research communities. In our architecture, on the other hand, ER and decision heuristics play a key role in guiding reasoning and learning.

**Figure 4:**
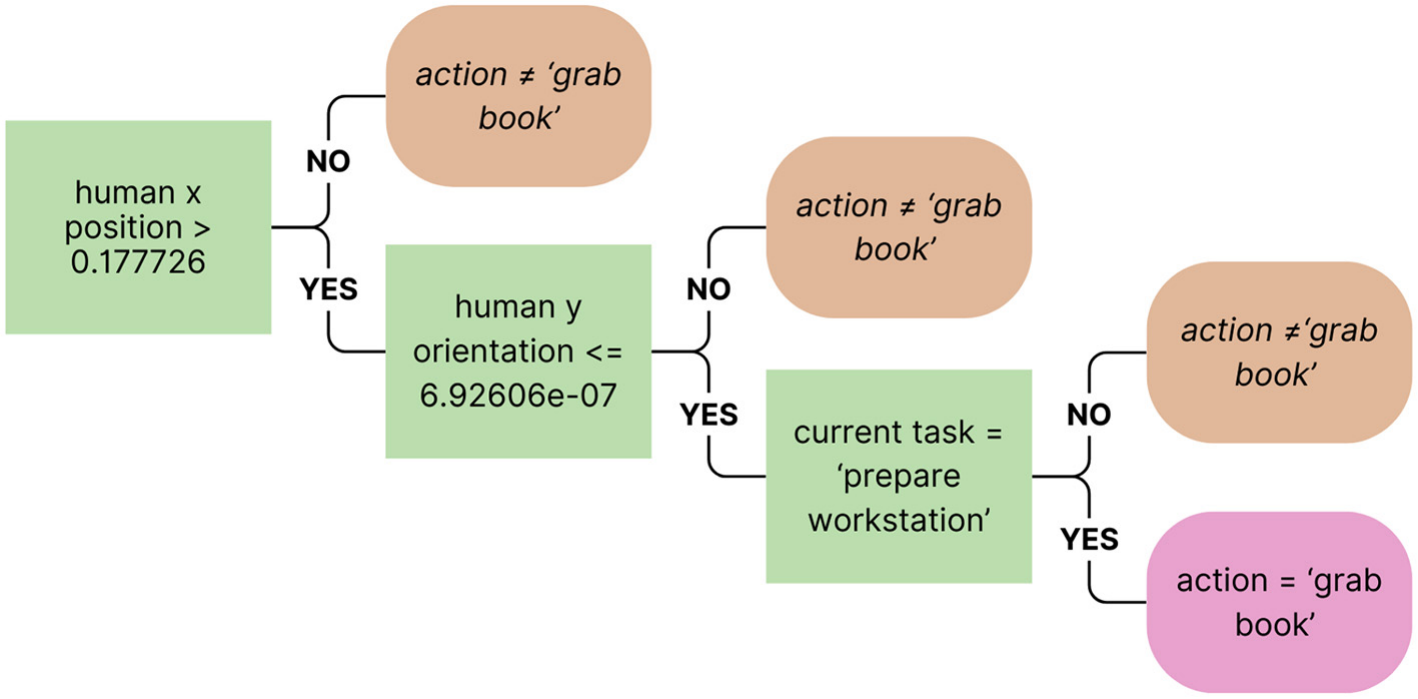
FF tree model of human behavior for the grab_book action in the example scenario from the *VirtualHome* domain.

Specifically, KAT enables the *ad hoc* agent to select relevant attributes and learn models of the other agents behavior incrementally and from limited data. The agent learns an ensemble of *Fast and Frugal* (FF) trees to predict the behavior of each teammate (or type of teammate). Each FF tree provides a binary choice for a particular action, and the number of leaves in a tree is limited by the number of attributes (Katsikopoulos et al., 2021). Each level of the tree contains an exit, allowing the agent to make quick decisions based on available data. Unlike many sophisticated methods for AHT, these predictive models can be learned and revised incrementally and rapidly. Also, the *ad hoc* agents make decisions efficiently by evaluating the more informative attributes and stopping as soon as a rational option is found. Figure 4 shows an FF tree learned for a human, with Table 1 showing the key attributes used in these trees. These trees are built to minimize false positives, with the initial version based on only 1, 000 traces of other agents' action choices and domain states. Since each FF tree provides a binary choice, we build each predictive model as an ensemble of FF trees with a simple decision tree choosing an action based on the output of the FF trees. As we show later (Section 4.2), reasoning with prior knowledge and these models provides much better performance than methods that just reason with knowledge or just use much larger learned models.

**Table 1:** Attributes used to create the behavior models of the other agents in VirtualHome.

Description of the attribute
Immediate two previous actions of the agent
Position of the agent (x,y,z)
Orientation of the agent (x,y,z)
Objects associated with the goal
Any objects in the hand of the agent
Any objects in the hand of the remaining agents
Current and previous tasks
Flags (weekday, going to office, and guests expected)

Consistent agreement (or disagreement) between actual observations (of outcomes) and the predictions provided by these behavior models triggers the use of a particular model for subsequent steps, or leads to the revision of the existing model(s), allowing the *ad hoc* agent to quickly adapt to changes in the domain or another agent's behavior over time.

### 3.3 Task anticipation

As discussed in Section 2, there is increasing evidence that LLMs (by themselves) are not a good choice for planning in practical domains, and that they make arbitrary decisions in novel situations (Lu et al., 2024). They have shown to be more effective when used as translators between natural and domain-specific languages (Guan et al., 2023), and to generate high-level (i.e., generic or abstract) guidance that is validated before being implemented by suitable planning subroutines (Kambhampati et al., 2024).

Motivated by these findings, KAT enables an *ad hoc* agent to use an LLM to anticipate the high-level future tasks (e.g., *prepare dinner*) in the domain based on the tasks executed so far or in similar situations. Recall that in the absence of the LLM, the agents are informed about the tasks to be executed one at a time. When the LLM is included in the architecture, each *ad hoc* agent anticipates the task likely to be assigned once the current task is done. By considering the current and anticipated tasks as joint goals, the *ad hoc* agent can prepare for upcoming tasks while completing the current task, e.g., fetch some ingredients from the fridge for making lunch while fetching eggs for cooking breakfast. We experimentally demonstrate in Section 4.2 that this strategy of targeting joint goals improves the team's performance.

Figure 3 shows an example input prompt to the LLM. Since LLMs are known to be sensitive to prompt formulation, we explored a combination of three prompting strategies in KAT:

1. **Adopting persona:** A specific role or character is assigned to guide the LLM's responses to be more (contextually) consistent with the assigned role.
2. **Few-shot prompting:** The prompt includes a few examples of the expected output in specific situations, guiding the use of pretrained knowledge.
3. **Chain-of-thought (CoT):** The prompt includes a step-by-step reasoning process that can be followed to arrive at an answer, leading to more accurate responses.

The “system message” (Figure 3) is used to guide the LLM to adopt the persona of a household assistant. It also instructs the LLM about the current objective, i.e., to complete the partially completed routine by selecting potential future tasks from the provided list of tasks in our example scenario within the *VirtualHome* domain. The “few-shot” prompting approach is used to include two task routines (chosen randomly from previous days) in the prompt. These routines can be at different stages of completion, e.g., one with no completed task and another with a partially completed routine. Next, CoT prompting is used to explain the reasoning behind each task in the few shot examples. For example, in Figure 3, a sample routine like “prepare breakfast weekday, prepare home workstation, prepare coffee, and prepare lunch” is explained with CoT method as “Since it is a weekday, breakfast needs to be prepared first to get the human ready for the day, after which the workstation should be set up to ensure the human can be productive during the day ...”. These explanations can be manually provided by the system designer or generated using the LLM itself. Finally, the system message, few-shot examples with CoT explanation, and the current query are combined and provided as the input prompt to the LLM.

When the LLM provides an output in response to the prompt, an *external validator* parses the LLM's output to check whether the tasks are feasible and in a reasonable order. Since LLMs are trained with large volumes of text from many domains, the anticipated tasks may not be feasible or may not match the actual needs in our domain. Thus, the validator uses domain-specific knowledge (and task priorities or preferences, if any) from previous experience (e.g., recent observations) to revise and reorder these tasks. Specifically, it filters historical data using domain-relevant contextual features (e.g., guests expected on Friday evening) and compares the filtered data with LLM's output of expected tasks. This helps the *ad hoc* agent eliminate tasks that are invalid or irrelevant, and reorder tasks according to the human preferences, e.g., in Section 4.2 the *ad hoc* agent prioritizes preparation of the workstation for work over packing a shopping bag during a weekday. The validator is intentionally simple, making only the minimal modifications necessary. These validated high-level anticipated tasks can serve as joint goals to be achieved. Since this list of validated tasks can change over time, KAT enables the *ad hoc* agent to consider one anticipated (future) task along with the current task, along with predicted actions of teammates over the next few steps, to plan a sequence of actions to be executed. We show in Section 4.2 that performance is much better with the LLM-based anticipation than without it.

### 3.4 Transparency in decision-making

An automated decision-making system's ability to answer questions about its decisions promotes acceptability (Anjomshoae et al., 2019; Fox et al., 2017); this transparency also plays an important role in human reasoning and learning. Unlike existing methods that seek to make a complex learned model interpretable, or to explain (or justify) all the choices made by a reasoning system, KAT seeks to respond to any given question about specific decisions made by the *ad hoc* agent or the human (teammate) by quickly identifying the relevant information and constructing relational descriptions.

The design choice of using knowledge-based reasoning and simple predictive models in KAT is the foundation for the approach described in this paper to provide the desired on-demand descriptions. We build on prior work in our group that demonstrated the ability to identify the axioms and literals relevant to the questions posed to a system making automated decisions (Sridharan, 2024). The underlying principle is that of *explainable agency*, which corresponds to enabling specific functional abilities (Langley, 2019; Langley et al., 2017). Specifically, the agent must be able to: (i) provide on-demand justification of decisions made during (or after) plan generation and execution by considering alternative choices; (ii) report actions executed and present information at a suitable level of abstraction; (iii) describe how actual events deviated from a computed plan and how it adapted to these deviations; and (iv) communicate information about decisions and justification such that it makes contact with human concepts such as beliefs and goals.

As described in Section 3.1, KAT is designed to represent and reason with knowledge at different abstractions. We build on this representation and the associated update processes to embed the principle of explainable agency, i.e., to implement the abilities mentioned above, enabling the *ad hoc* agent to generate relational descriptions in response to four types of questions identified as being important in work on explainable planning (Fox et al., 2017). Here we describe the procedure the *ad hoc* agent follows to generate responses for each of these types of questions. Examples of the use of this procedure to construct explanations on-demand are provided later in Section 4.3.

1. **(Causal questions)** *Why did you execute* *a*_*I*_*, i.e., action* *a* *at step* *I?*

- If *a*_*I*_ is not the last action of plan *P* executed by the agent, extract actions {*A*_*af*_}∈*P* that occurred immediately after *a*_*I*_∈*P*.
- Examine Π(D,H) to identify axioms with the negation of *a*_*I*+*i*_∈{*A*_*af*_} in *head*, i.e., axioms encoding conditions that prevent *a*_*I*+*i*_ from occurring.
- Check if each such axiom's *body* is satisfied by *answer set* at time step *I*. If yes, identify fluent literals *f* in *body* whose value changed from *I* to *I*+1.
- All such literals {*f*} over all identified axioms have been changed by the execution of *a*_*I*_. Collect these literals to construct the answer.
- If *a*_*I*_ is the last action in *P*, it contributed directly toward achieving the goal. Use goal *G* and *a*_*I*_ to construct the answer.

**2. (Contrastive questions)** *Why did you not execute* *a*_*I*_*, i.e., action* *a* *at step* *I?*

- Examine Π(D,H) to identify axioms with the negation of *a*_*I*_ in its *head*, i.e., executability conditions.
- Check if each selected axiom's *body* is satisfied by *answer set* at *I*. If yes, collect fluent literals {*f*} in *body* as they prevented consideration of *a*_*I*_.
- If not, examine Π(D,H) to identify axioms with *a*_*I*_ in its *body* alongside other literals {*f*′}, i.e., causal laws.
- Extract the additional precondition literals {*f*′}. Examine Π(D,H) to identify axioms with *l*∈{*f*′} in its *head*, i.e., state constraints.
- Check if selected axiom's *body* is satisfied by *answer set* at step *I*. If not, use literal *l*∈{*f*′} and the *body* literals of axiom to construct answer.

3. **(Justify beliefs)** *Why did you believe* *l*_*I*_*, i.e., l at step I?*

- Replace the grounded terms of *l*_*I*_ with the corresponding variables.
- Examine Π(D,H) to identify axioms with *l*_*I*_ in *head*. i.e., state constraints.
- Check whether each selected axiom's *body* is satisfied by the answer set at step *I*. If yes, collect fluent literals {*f*} in *body* as they support belief *l*_*I*_.
- If multiple axioms are identified, select one of them to provide explanation.
- If belief tracing is enabled, create a tree with *l*_*I*_ as its head and each selected axiom as a branch. With the axiom, also store the supporting {*f*} fluents.
- Repeat procedure for each fluent literal in {*f*} as target belief until no more axioms are identified.

**4. (Counterfactual questions)** *What will be the outcome if you (or human* *R) execute* $a_{I}^{\prime }$ *in (future) step* *I? What will be the resultant world state at (future) step* *I* *if the human executes actions predicted by learned models?*

- Retrieve most recent state observation of environment *S*_*I*−*n*_ in relation to step *I*. i.e., start with the current best estimate of world state.
- Use the predictive behavior models of human(s) to retrieve their action $\left\{A_{I-n}^{ot}\right\}$ for step *I*−*n*. KAT provides action for the *ad hoc* agent, *a*_*I*−*n*_.
- Perform mental simulation of the future step *I*−*n*+1 from *S*_*I*−*n*_ using existing knowledge and action choices of human(s) $\left\{A_{I-n}^{ot},a_{I-n}\right\}$.
- Repeat above *n* times, i.e., roll out the future and explore effects of the action of *ad hoc* agent and human until environment reaches the queried step *I*. Collect the state information *S*_*I*_ at *I*.
- Feed *S*_*I*_ to the predictive behavior model of the human *R* (KAT) to retrieve the action $a_{I}^{o}$ (*a*_*I*_) for *R* (*ad hoc* agent) in state *S*_*I*_.
- Traverse through the FF tree model of *R* to identify active branches when selecting $a_{I}^{o}$. Collect $a_{I}^{o}$ and these branches to construct the answer.
- Replace action $a_{I}^{o}$ (*a*_*I*_) with $a_{I}^{\prime }$ for the human *R* (*ad hoc* agent).
- Roll out environment one step to obtain the resultant state *S*_*I*+1_. Collect state information *S*_*I*+1_ to construct the explanation.

The acquired information may be used for further training, especially the human behavior prediction models. For all types of questions, the identified literals are processed using existing tools and templates to generate textual descriptions provided as responses (i.e., explanations) before, during, or after planning or execution. Section 4.3 provides execution examples.

### 3.5 Knowledge acquisition

In practical domains, agents often do not have comprehensive knowledge, and making decisions based on incomplete knowledge leads to ineffective collaboration. KAT enables an *ad hoc* agent to incrementally acquire missing domain knowledge, reduce ambiguity, and support more reliable decision making. It does so by embedding the principle of *interactive learning*, which jointly refers to different types of learning such as supervised, unsupervised, and learning from reinforcement (Laird et al., 2017). The difference lies in how this learning is achieved. As discussed in Section 2, modern AI systems focus on building a single large model or policy with many free parameters for different situations, platforms, and/or application domains. The learned model or policy is then hard to understand or revise rapidly, making them unsuitable for decision making under true open-world uncertainty (Katsikopoulos et al., 2021). Our interpretation of interactive learning, on the other hand, focuses on learning as needed to adapt to any given domain and set of tasks, reasoning with prior knowledge and decision heuristics to trigger and constrain the learning, and using the learned knowledge for reasoning. It also enables cumulative learning through memory consolidation, revising the learned knowledge and discovering high-level (i.e., more abstract) concepts and theories to update the existing knowledge (Stickgold, 2005). Such an approach is essential for knowledge acquisition, information storage, and information retrieval in humans (Baddeley, 2012), and it leads to simpler models amenable to rapid revisions.

Specifically, KAT supports two strategies for the agent to acquire knowledge. First, the agent learns from cues provided by a human during task execution—Algorithm 1 (Section 3.5.1); second, it refines this knowledge by exploring the newly learned actions in different initial states, identifying and correcting inconsistencies (Section 3.5.2).

Algorithm 1 Acquire knowledge from human cues. 
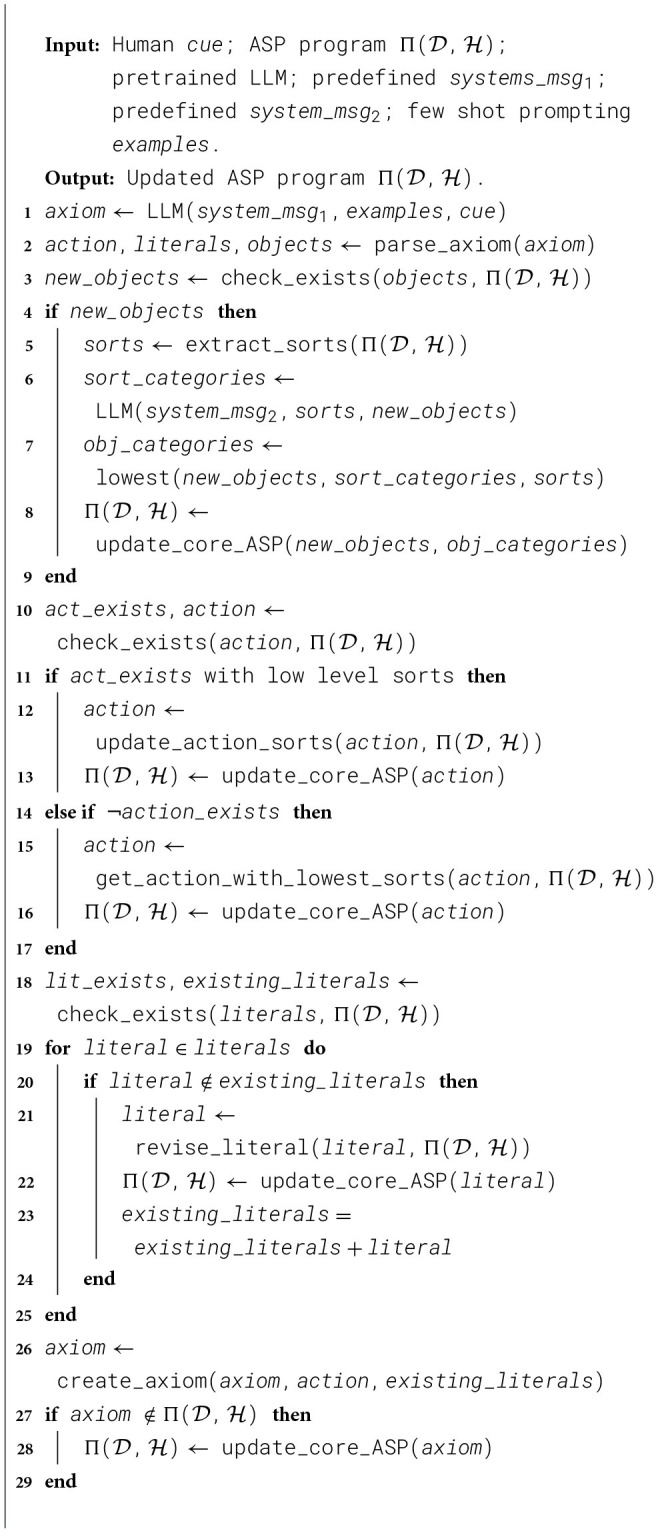

#### 3.5.1 Learning from human cues

**Figure 5:**
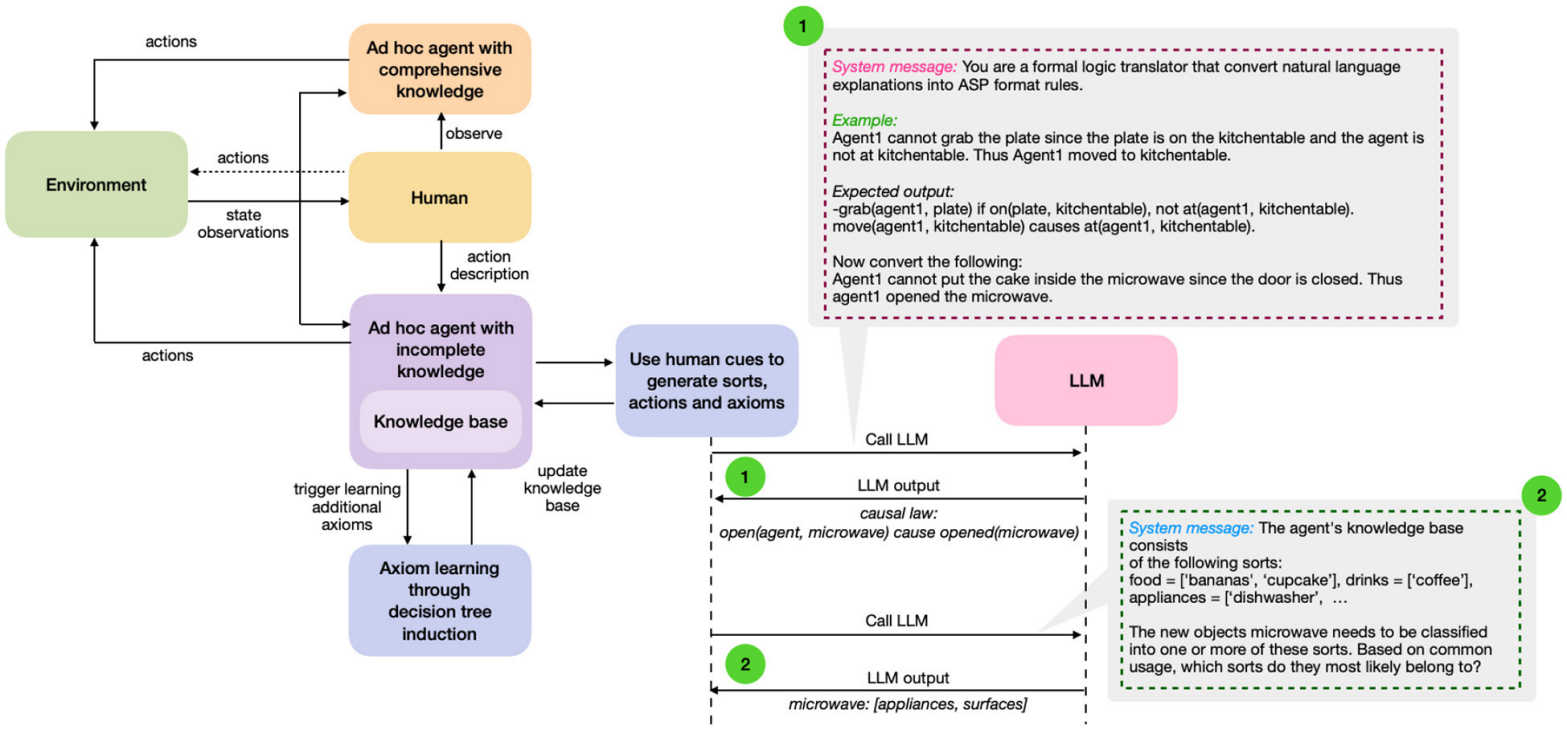
KAT leverages LLM, decision tree induction and prior knowledge to map natural language descriptions of actions and unexpected outcomes into new objects, actions and axioms in non-monotonic logical reasoning.

The process of generating human cues in our experimental evaluation is described further in Section 4.1. This cue describes capabilities that the *ad hoc* agent is previously unaware of. When an *ad hoc* agent receives such a cue from the human, e.g., “Agent 1 cannot put the cake inside the microwave since the microwave's door is closed,” it automatically generates and sends a prompt to the LLM by combining a predefined system message (message 1 in Figure 5) with two examples of input and expected output, the structure of the three types of axioms (e.g., “*a* causes *f* if *p*”), and the query based on the cue (Line 1, Algorithm 1). The LLM maps this cue into candidate axioms using their known structure. The LLM's output is parsed using regular expressions to discard any output that does not match the expected format of axioms. From the LLM's output, the agent extracts actions (verbs), objects, and action preconditions and effects (Line 2). The agent then checks whether the new objects are already defined in the current ASP program (Π(D,H), Line 3). Any new objects are returned to the LLM with a second prompt (message 2 in Figure 5) based on a predefined message template, existing sorts in Π(D,H), and the new objects (Lines 4–6). The LLM categorizes each new object into one or more known basic sorts. If multiple sort labels are returned for an object, the lowest category in sort hierarchy is used to add the new object to the program Π(D,H) (Lines 7–8).

Next, the agent examines Π(D,H) to check whether the action verb (e.g., *grab*) from the LLM's output is already defined (Line 10). If an action that semantically matches this action verb exists in Π(D,H), the agent retrieves it; this action may exhibit the same behavior as the one extracted from the cue but have a different name, e.g., *pick(A,O)* instead of *grab(A,O)*. The agent performs strictly controlled verb synthesis with WordNet (Miller, 1995) to retrieve synonyms for the action verb from the cue and checks the synonyms with the Σ of Π(D,H). If a match is found, the action verb from the LLM output is replaced by the existing action in Σ.

Also, the sorts of the arguments of the action extracted from the human cue may differ from those of the action in Π(D,H), e.g., they may be sub-sorts or parent sorts. If the sorts of arguments of the action in Π(D,H) are parent sorts of those in the cue, the agent uses the existing action as is; if not, the agent lifts the sort of the existing action's arguments to the new sorts and updates Π(D,H) (Lines 11–13). If the extracted action (and any equivalent) does not exist in Π(D,H), the agent adds it with the lowest (i.e., most specific) sort labels for arguments (Lines 14–17). This process ensures that we do not introduce the same action multiple times with different sorts as its arguments. This procedure is also repeated for literals (Lines 18–25), and the actions and literals are used to convert the extracted axioms to the appropriate format, e.g., “*a* causes *l*” converted to “*holds(f,I+1) :- occurs(a,I)*”, replacing the ground sorts with variables and ensuring consistency between head and body of the axiom (Line 26). The axiom is then added to Π(D,H) if it does not exist (Lines 27–28).

#### 3.5.2 Learning from observations

The second strategy enables the *ad hoc* agent to refine its knowledge based on observations obtained while using the existing knowledge for planning and execution. It extends and adapts existing ideas on combining *decision tree induction* with knowledge-based reasoning (Mota et al., 2021).

1. The agent selects an action *a*_*I*_ from the newly learned set of actions *A* and an initial state of the environment *S*_*I*_. It simulates the execution of *a*_*I*_ in *S*_*I*_ to collect information about the outcomes (e.g., end state, presence, or absence of inconsistency).
2. The absence of an expected outcome (i.e., an inconsistency) indicates the potential absence of an executability condition; any additional effects indicate missing causal law(s). If all observations match expected outcomes for different actions and initial states, the current knowledge is considered to be comprehensive.
3. The agent responds to an inconsistency by simulating the execution of *a*_*I*_ in different states and stores all fluent literals in the answer set or initial state, with an object constant that is in *a*_*I*_. These collected literals form part of the *training* examples.
4. In the *training* examples, the ground terms in literals are replaced by variables, and the dataset is reformatted with the fluent literals as features and the presence or absence of inconsistency as the class label. Each training example then records the occurrence or non-occurrence of a fluent literal, and the presence or absence of an inconsistency, as a binary value.
5. Separate decision trees are constructed for causal laws and executability conditions, with the action as the root node, and nodes are split using features that have not been used before and are likely to result in the highest reduction in entropy.
6. Candidate axioms are generated by traversing the learned trees from the root to the leaves using only those nodes that agree with their class label up to a threshold level (90%) and contain at least a minimum percentage (2%) of the dataset.
7. Only candidate axioms that have sufficient support among the training examples (90% in our experiments) are retained. Also, the decision tree induction process is repeated multiple times over the training data to explore different subsets of data. Only axioms that are retained over multiple such repetitions are lifted to the more general form and added to Π(D,H).

**Figure 6:**
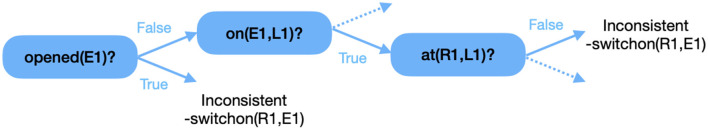
Part of the decision tree created to learn missing executability conditions.


Figure 6 shows part of a decision tree generated by this process, leading to learning the following two executability conditions by the *ad hoc* agent:

$$\begin{array}{ll} -occurs\left(switchon\left(R,E\right),I\right)\leftarrow & holds\left(on\left(E,L\right),I\right), \end{array}$$ (7)

$$\begin{array}{l} notholds\left(at\left(R,L\right),I\right),loc\left(L\right),\\ agent\left(R\right),appliance\left(E\right). \end{array}$$

$$\begin{array}{ll} -occurs\left(switchon\left(R,E\right),I\right)\leftarrow & holds\left(opened\left(E\right),I\right), \end{array}$$ (8)

$$\begin{array}{l} agent\left(R\right),appliance\left(E\right). \end{array}$$

which imply that an agent cannot switch on an appliance if it is not in the same location or if the appliance's door is open. Although the learning strategy is described above in the context of learning new objects, actions and axioms, it can be used to learn new literals (relations) as well.

### 3.6 KAT control loop

The overall control loop of KAT is described in Algorithm 2. First, the *VirtualHome* environment is set up, including the other agents' policies P and the tasks T that need to be completed within the given day (Line 2); both P and T are unknown to the *ad hoc* agent. This will decide the state s of the environment. Next, the first task of the day is assigned to the agent team for joint execution (Line 5). The *ad hoc* agent also used the procedure described in Section 3.3 to anticipate the future tasks in the domain (Line 7). The other agents' actions are identified based on the current state, task, and P (Line 8). The *ad hoc* agent's action is computed by reasoning with domain knowledge and learned models (M) of other agents' behaviors while considering both current and future tasks as joint goals (Line 9; Algorithm 3). The selected actions are then executed in the simulated environment to receive the updated state (Line 11). The observed state information used to incrementally revise M, e.g., when the observed and predicted behaviors do not match (Line 12). The updated state is used for subsequent reasoning and task execution (Line 13). This process is continued until all the tasks in T are completed successfully.

Algorithm 2 Control loop. 
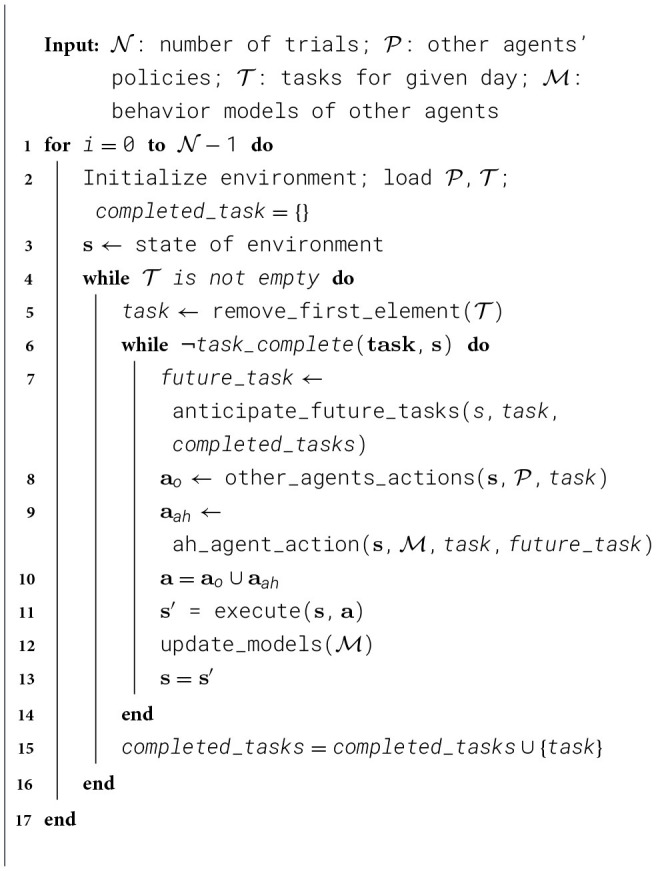

Algorithm 3 describes how the *ad hoc* agent computes its action in Line 9 of Algorithm 2. This agent first uses the learned behavior models (M) of the other agents to predict their next action in the current state (Line 1). It mentally simulates the effect of these action to estimate the next state (Line 2). While Algorithm 3 demonstrates this mental simulation for one time step, this process can be repeated over multiple steps to predict and reason about the other agents' actions over multiple time steps. Given the complexity of the *VirtualHome* domain, with its many objects, appliances, and locations, considering the entire state information would significantly increase the computational complexity of the reasoning process. Thus, the *ad hoc* agent uses its knowledge of the current state, predicted action(s) of other agents, predicted future state(s), and current and anticipated task(s) to compute the relevant signature (e.g., specific objects and locations in the domain) that need to be considered (Line 3); this process is similar to the zoom operation described in Section 3.1. For example, if a task does not involve any objects from the bedroom, information related to that location (and the objects in it) can be excluded from the current decision-making process. Similarly, any other objects unrelated to the current task can be disregarded. This information is used to automatically determine the level of abstraction, and the information at that abstraction, to be used for reasoning. The relevant information from the core ASP program (without description of current state and observations) and the goal are used to automatically construct the ASP program to be used for reasoning (Lines 4–5). The corresponding answer set obtained by solving this ASP program provides the next action(s) to be executed by the *ad hoc* agent (Lines 6–7), which is returned to Algorithm 2.

Algorithm 3 Compute *ad hoc* agent action. 
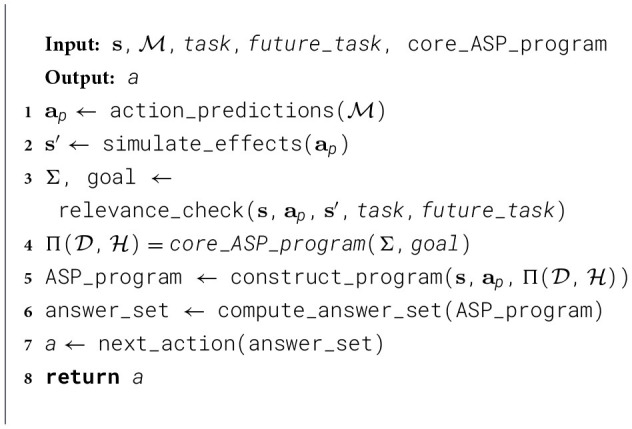

## 4 Experimental setup and results

We experimentally evaluated the following hypotheses regarding KAT's capabilities:

**H1** Reasoning with prior knowledge and the rapidly-learned behavior prediction models improves performance and promotes scalability;
**H2** Using LLM-based anticipated tasks as joint goals improves performance compared with planning for one task at a time;
**H3** Incrementally-updated prompts and validators improve task anticipation capability of the LLM and the team's performance;
**H4** Using the LLM to directly output a sequence of low-level actions to complete assigned tasks results in poor performance;
**H5** KAT enables the *ad hoc* agent to accurately learn previously unknown objects, actions, and axioms;
**H6** Reasoning with incrementally learned knowledge improves the performance of the *ad hoc* agent and the team; and
**H7** KAT enables the *ad hoc* agent to generate relational descriptions as explanations of its decisions and beliefs and those of the other agents.

We evaluated these hypotheses in the *VirtualHome* domain, where each episode involved AI *ad hoc* agent(s) and a human agent collaborating to complete household tasks. We recorded the number of steps (plan length) and the total time taken to complete the task(s) as the *performance measures*. Note that the total time here corresponds to the time taken to execute the selected actions in the *VirtualHome* domain and does not include planning or other computation times. Additional details about experiments, baselines, and measures are provided below. All prompts to the LLM were through OpenAI GPT4o mini API with default parameters (e.g., temperature = 1.0).

### 4.1 Experimental setup

We seek to demonstrate and evaluate the ability of each *ad hoc* agent (equipped with our architecture) to model and reason about the behavior of other (AI, human) agents with a range of capabilities and preferences, with the longer-term goal of having AI agents and robots assisting humans in practical environments. It would have been infeasible to involve an actual human in our experiments while still conducting a sufficient number of trials to draw statistically significant conclusions. So, similar to other work in human-agent or multiagent collaboration, we had to: (i) consider a range of different preferences and capabilities for other agents, with these preferences and capabilities being unknown to the *ad hoc* agent(s); and (ii) develop a mechanism to encode these preferences and capabilities such that we could automate the generation of the behavior of the different agents that our *ad hoc* agent(s) interacted with during experimental trials.

Some of the preferences and capabilities we considered were explicitly those of a human living in a home and being assisted by AI agents. This included the preference of a particular beverage (coffee vs. tea) at a particular time of day, or a particular breakfast (e.g., toast and cereal). It also included action capabilities such as sitting in a chair and working on a computer. Hence, we called such agents *human* agents. None of the agents knew the preferences or capabilities of the other agents initially.

To automate our experimental trials, we then used ASP programs to encode the preferences and capabilities of the (AI, human) agents interacting with our *ad hoc* agent(s) in the simulation environment. The ASP program for the simulated human did not have access to any models predicting the teammates' actions. Each *ad hoc* agent, on the other hand, built models that predicted the behavior of the other agents based on limited observations of their action choices, as described in Section 3.2. This does not, however, imply that the behavior of the human (or other AI agents) is fully deterministic. The ASP programs of the agents that are not *ad hoc* agents introduce noise in the outcome of specific actions executed by these agents, and the execution of some actions (in the simulation environment) by any agent sometimes does not lead to the expected outcome. Furthermore, none of the agents have comprehensive knowledge of the environment, which also leads to unforeseen circumstances; this is the motivation for the knowledge acquisition component of our architecture (Section 3.5).

The task generator provided a sequence of tasks for any given day. These sequences involved realistic, complex household tasks such as *prepare breakfast, prepare home-workstation, make coffee*, and *prepare lunch*. Completing each task required multiple interdependent actions involving finding and retrieving objects from specific locations; placing objects in target locations; interacting with appliances by opening or closing them; and turning appliances on or off for cooking, cleaning, and other purposes. The sequence of tasks generated by the task generator were assigned to the agents over time. Recall that the generator produced these tasks in a realistic order respecting the dependencies among the tasks (e.g., breakfast is prepared before coffee). The agents were not aware of the complete sequence and received one task at a time. The human was assigned the same goal as the *ad hoc* agent(s). All agents received the same observations of the domain at each step, which they used to plan their respective actions. There was no direct communication between them.

When an *ad hoc* agent equipped with KAT received a task, it prompted the LLM—see Section 3.3 and Figure 3. The anticipated tasks were validated and mapped to ASP literals, with the next anticipated task and current task set as joint goals for this agent. During planning, the *ad hoc* agent also used the learned behavior prediction models to predict each teammate's actions for a few steps—see Section 3.2. Recall that these predictive models were built using just 1, 000 examples of prior traces of actions and domain state of a few different hand-coded behaviors. The *ad hoc* agent initially assigns one learned model to each teammate but uses information from subsequent steps to incrementally revise models for each teammate based on their observed behavior. Predicted actions from the models were then mapped to exogenous actions that were added to the ASP program along with initial state information and refined sorts. The ASP program of the *ad hoc* agent included additional axioms for reasoning about these predicted actions of each teammate. As a result, the *ad hoc* agent's plan anticipated preconditions of some intermediate steps to be satisfied by a teammate's actions, even though the teammate did not always execute that action. The *ad hoc* agent hence had to respond to unexpected action outcomes and domain states.

In **Exp1**, we randomly selected 100 task routines sampled from predefined sequences and measured the ability of a team comprising a human and an *ad hoc* agent to complete these tasks. Performance measures were the number of steps and time taken to successfully compete all the tasks assigned for a given day. We used three baselines:

- **Base1**: used LLM for anticipating future tasks, but did not use models to predict human's behavior.
- **Base2**: did not use LLM to anticipate future tasks, but used models to predict the human's behavior.
- **Base3**: did not use LLM for task anticipation or behavior models to predict human's actions.

We chose these baselines because we could not find any existing framework for AHT that supported all the capabilities of our architecture. This choice also allowed us to conduct ablation studies that experimentally evaluated the contribution of each key component of KAT. The results from these experiences were used to evaluate **H1** and **H2**.

Since the actual time taken and the number of action steps required to complete tasks can vary substantially based on the tasks under consideration, the average of these values over the individual trials may not be meaningful. We instead *ran paired trials and computed the performance measure values for the baselines as a fraction of these values for KAT in each trial*. We then reported the average of these ratios.

For evaluating scalability in **H1**, we increased the team size by introducing additional *ad hoc* agents, with three agents (one human, two *ad hoc* agents) and four agents (one human, three *ad hoc* agents) collaborating to complete tasks (as in **Exp1**). These different configurations would normally make collaboration increasingly challenging, e.g., with just two agents (one *ad hoc* agent, one human) the domain has ≈10^25^ possible states, and this number increases exponentially with the number of AI agents. We then measured the number of steps and time taken by the agent teams to complete the tasks.

Next, in **Exp2**, we computed the precision and recall of the tasks anticipated by the LLM, before and after applying the validator, over the 100 task routines. We also computed the precision and recall of a simple statistical model that replaced the LLM and anticipated the agent's next task based on past experience.

In **Exp3**, we randomly selected 20 task routines and recorded the team performance (number of steps in the computed and executed plan; task completion time) when the LLM used the combination of prompting methods (Section 3.3) and when it did not. We used four baselines:

- **Base4**: none of the prompting strategies or validator.
- **Base5**: few-shot prompting but no external validator.
- **Base6**: CoT prompting but no external validator.
- **Base7**: external validator but no prompt-engineering.

The results from these experiments were used to evaluate **H3**.

For evaluating **H4**, we conducted a special experiment, **Exp4**, in which we created an architecture that used the LLM for directly computing sequences of actions for specific tasks (**Base8**). Specifically, our prompt included details of actions available in our example scenario in *VirtualHome*, their intended purpose [from ASP program, e.g., move(agent, location): move the agent to an adjacent location; grab(agent, object): pick up an object]. We also supplied the LLM some **Action Feasibility Rules**:

- *Movement limitation* (critical): must only move to adjacent locations defined by the next_to relationships. Always check adjacency before predicting a move.
- *Object location*: must be in the same location as an object to act on it (e.g., grab, put).
- *Carrying limit*: cannot hold more than two objects. When holding two objects, actions like open, close, switch-on, or switch-off require you to put at least one object down first.
- *Appliance safety*: for safety, you should not open appliance doors when they are switched on.
- *Avoid conflict*: If the human is holding an object, they will handle all actions with the object. Do not attempt to grab or interact with this object. Instead, focus on other parts of the goal.

We included information about adjacent places in the domain, emphasizing the fact that the agent can only move between the defined adjacent places.

The LLM also had access to the current world state, including the location of the agents, objects, and appliances, each appliance's state, and information about the objects held by the agents. The problem specification also described the task to be performed; the immediate previous actions of the human and the *ad hoc* agent; any specific information to be considered on any given day (e.g., human working from home; whether it was a weekday or the weekend; and whether the human was expecting guests). In addition, the prompt included a detailed example of selecting an action, and asked the LLM to generate an action sequence for achieving the assigned goal and specify the next action to execute.

The LLM's action choice was assigned as the *ad hoc* agent' action. As a recovery mechanism, we corrected errors in the LLM output up to three times per trial. For example, if the LLM's action involves grabbing an object without moving to the appropriate location, we provided feedback explaining why this choice was incorrect and allowed the LLM to predict another action for that step. We then measured the performance of the human-*ad hoc* agent team to complete the previously selected 100 task routines. As before, the performance measures were the number of steps and time taken to complete the set of tasks.

To evaluate **H5**, in **Exp5**, we introduced another *ad hoc* agent with an incomplete knowledge base to the two agent team (*ad hoc* agent and human). The new agent's ASP program included only a subset of the objects (17/31), actions (4/7), and axioms (6/9 causal laws, 16/26 executability conditions). This corresponded to the absence of around 40%−45% knowledge. We made sure that this agent had enough initial knowledge to perform some basic activities, while also withholding key knowledge to create gaps that limited the agent's ability to complete tasks. The process for automatically generating human cues mimicked a human providing feedback that enables the *ad hoc* agent to acquire knowledge about other agents. During task execution, the simulated human agent periodically described actions of the knowledgeable *ad hoc* agent to the *ad hoc* agent with missing knowledge. This was achieved by a script (Python program) that considered a preset (natural language) template for such cues, the current state, and the action executed or rejected by the knowledgeable agent, e.g., “Agent < *A*> < *can*/*cannot*> execute action < *X*> on object < *Y*> because < *Z*>.” The variables in the template were ground at run-time using knowledge of the current state (obtained from the simulation environment and mapped to domain literals), and preconditions and effects of actions encoded in relevant ASP programs. The *ad hoc* agent (with missing knowledge) then used the procedure described in Section 3.5 to process these descriptions into ASP sorts, actions and axioms with the help of an LLM and added the validated information to its knowledge base. At the end of each episode, it also used decision tree induction to learn new axioms. We then evaluated the *ad hoc* agent's ability to learn missing knowledge across 10 episodes. Similar to previous experiments, task sequences were generated by the task generator and provided to the agents one at a time. However, in these experiments we intentionally omitted the future task anticipation algorithm to prevent its influence on knowledge acquisition capabilities. We then recorded the number of objects, actions, and axioms learned in each trial, and the precision and recall of learning these axioms compared with a complete ASP program (ground truth).

Next, in **Exp6**, we extended each episode from **Exp5** to include three consecutive runs. The first run in an episode had the same initial knowledge as in **Exp5**, but the subsequent two runs built on the knowledge for a potentially different sequence of tasks. This process was repeated for the 10 episodes; we recorded the objects, actions, and axioms learned after each run and episode, and computed precision and recall as the performance measures.

To evaluate **H6**, in **Exp7**, we ran 20 trials with and without the learned axioms, and recorded the number of steps (plan length) and the time taken by the team to complete the assigned task(s).

Finally, to evaluate **H7**, we designed **Exp8** in which we randomly selected 10 configurations (from the 100 in **Exp1**) and saved the corresponding answer sets (with KAT, baseline) to provide ground truth. Then, we posed 32 different questions (divided between the four types of questions) about some chosen steps in each trial corresponding to one of these 10 configurations, with answers computed as described in Section 3.4. We recorded the precision and recall of retrieving literals to answer these questions, with saved answer sets used to provide ground truth. Furthermore, we considered execution traces as qualitative evaluation of **H7**.

### 4.2 Experimental results

Table 2 summarizes the results of **Exp1**. When the *ad hoc* agent reasoned with anticipated tasks and predicted human (agent) actions, it provided the best observed performance with the lowest number of action steps and least amount of time taken to complete the routines. The prediction accuracy of the behavior models learned by the *ad hoc* agent for the human using the ensemble of FF trees was 85%. We observe that the model does make errors, but it supports rapid learning and revision. Also, reasoning with prior knowledge and the output of these predictive models significantly improves the performance. When the *ad hoc* agent used task anticipation but did not use the behavior prediction models (**Base1**), the number of steps and time taken to complete tasks increased. This is due to the *ad hoc* agent's inability to predict its teammate's actions causing it to execute the same actions as a teammate, hindering collaboration. These results emphasize the importance of using the behavior prediction models, supporting hypothesis **H1**.

**Table 2:** Average number of steps and time taken to complete task routines, with values for baselines computed as a fraction of these values for KAT in each trial; for comparison, the average absolute values are 26.8 steps and 361 seconds for KAT.

Architecture	Steps	Time(s)
KAT (anticipate tasks, predict actions)	1.0	1.0
Base1 (anticipate tasks)	1.1	1.1
Base2 (predict actions)	1.3	1.2
Base3	1.4	1.4

Task anticipation (LLM) and teammates' behavior/action prediction (FF trees) substantially improved performance.

When the *ad hoc* agent used the behavior models to predict the human's actions, but did not anticipate and plan for future tasks (**Base2**), the performance worsened, with a further increase in the number of steps and the time taken to complete the tasks. Recall that this setting corresponded to the agent only planning for one goal at a time without anticipating future goals. Planning actions jointly for the current task and the anticipated (next) task saved time and effort. For example, when the agent visited the bedroom to retrieve a board game for the guests, it also picked up bottles of wine from the cellar that is on the way. Making two separate trips for these tasks extended the length and duration of the plans. These results indicate the impact that planning for joint goals has on performance, supporting **H2**. When the *ad hoc* agent did not use task anticipation or the behavior prediction models (**Base3**), it resulted in the worst observed performance with the highest value for number of steps and time taken to complete the tasks. All results were statistically significant with *p* < 0.0001 These results support **H1** and **H2**.

Next, Table 3 summarizes the performance of **Team1** (human, one *ad hoc* agent), **Team2** (human, two *ad hoc* agents) and **Team3** (human, three *ad hoc* agents) in completing the same set of 100 task routines. As the number of *ad hoc* agents increases, task completion becomes more efficient: Team2 outperformed Team1 by requiring fewer steps and less time to complete the tasks, while Team3 showed further improvements over Team2. Moreover, the average time taken to generate a plan for a given goal (that consisted of 1–20 steps) remained consistent across all three teams, with 0.44 seconds for Team1, 0.49 seconds for Team2 and 0.45 seconds for Team3. These results emphasize the importance of efficient collaboration among agents and demonstrate the scalability of the architecture to multiple agents, further supporting **H1**. Once again, all the results were statistically significant with *p* < 0.0001. The observed performance was primarily because the design choices in our architecture enable each *ad hoc* agent to reason independently and efficiently using domain knowledge and learned models.

**Table 3:** Average number of steps and time taken by Team1 (human, *ad hoc* agent), Team2 (human, two *ad hoc* agents), and Team3 (human, three *ad hoc* agents) to complete the task routines, with performance measure values for Teams 2–3 computed as a fraction of the values for Team 1 in each trial; results indicated collaboration between agents leading to improved performance.

Team	Steps	Time(s)
Team1	1.0	1.0
Team2	0.8	0.9
Team3	0.7	0.8

Table 4 shows the results from **Exp2**, where we computed the precision and recall values of the tasks anticipated by the LLM before and after applying the validator. We observed marked improvement in precision after applying the validator to the LLM's output. The errors in the LLM's outputs were substantially reduced by the validator. The recall values did not change substantially as the validator did not introduce new tasks; it only reordered the tasks that are out of order and removed irrelevant tasks. On the other hand, the simple statistical model that anticipated future tasks based only on past experience resulted in precision and recall of 0.75 and 0.76 respectively. These results indicate that using just the historical data is insufficient for task anticipation. Instead, using the validator to correct the LLM's output based on prior knowledge and experiences leads to better results, supporting **H3**.

**Table 4:** Precision and recall values of the anticipated tasks with and without the LLM and the validator. A simple statistical model is comparable with an LLM, and the validator plays an important role in improving precision.

Architecture	Precision	Recall
Simple statistical model	0.75	0.76
LLM without validator	0.78	0.76
LLM with validator	0.99	0.78

Table 5 shows the results from **Exp3**, which explored the use of different prompting strategies and the validator (Section 3.3) with the LLM to anticipate future tasks. We again observed a significant improvement in performance, e.g., a lower number of steps and time to complete tasks, with the external validator and a combination of prompting methods. In particular, using the validator to adapt the LLM's output to the domain had a significant impact on performance, further supporting **H3**. These results were statistically significant for Base4, Base5 and Base6 with *p* < 0.001. For Base7 the results were mixed; the reduction in steps was statistically significant (*p* < 0.05), while the reduction in time was not (*p* = 0.09). This outcome is consistent with expectations since Base7 used the validator; it emphasizes the importance of the validator and the need for further exploration of more complex scenarios to determine the importance of the prompting strategies.

**Table 5:** Average number of steps and time taken by the team (human, *ad hoc* agent) to complete tasks with prompting strategies and/or external validator; performance measure values for baselines computed as a fraction of values for KAT in each trial, with the average absolute values being 27.5 steps and 372.7 seconds for KAT.

Architecture	Steps	Time(s)
KAT (all prompting, with validator)	1.00	1.00
Base4 (no prompting, no validator)	1.21	1.15
Base5 (few-shot prompting, no validator)	1.17	1.18
Base6 (chain-of-thoughts, no validator)	1.16	1.16
Base7 (no prompting, with validator)	1.05	1.04

Next, Table 6 shows the results of **Exp4**, where we used the LLM to directly compute the low-level actions for the *ad hoc* agent in the *VirtualHome* domain. The number of steps and time taken to successfully complete the task routines are significantly higher than KAT as well as all other baselines (**Base1-3**) in Table 2. Again the results were statistically significant with *p* < 0.0001. These results support **H4**.

**Table 6:** Average number of steps and time taken by the team (human, *ad hoc* agent) to complete task routines when the LLM directly outputs a sequence of low-level actions to be executed.

Architecture	Steps	Time(s)
Base8 (LLM predict low-level actions)	1.5	1.5

Performance measure values computed as a fraction of values obtained with KAT in Table 2.

**Figure 7:**
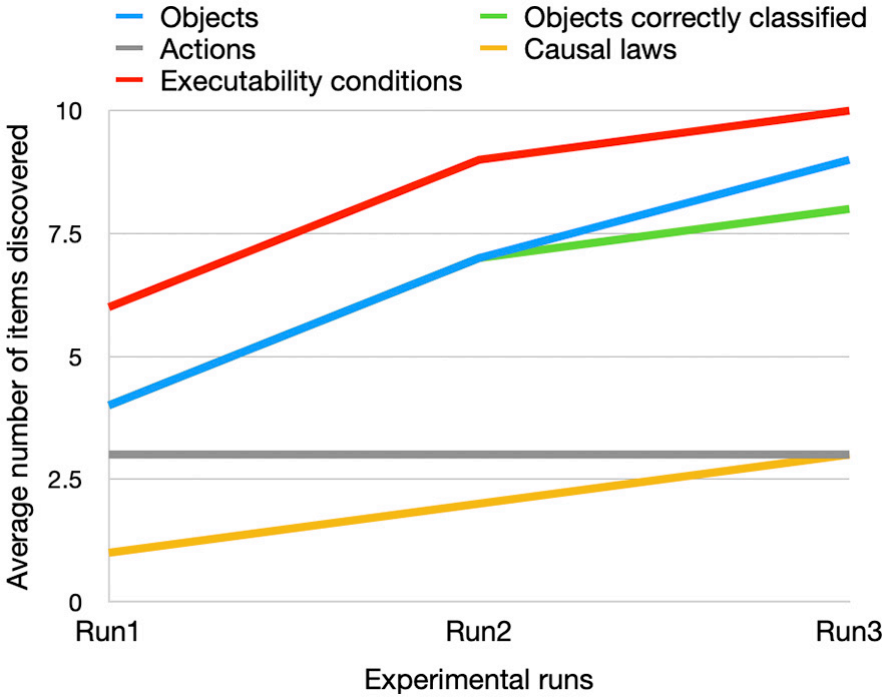
Average number of objects, actions, and axioms learned in three consecutive runs. Results averaged over 10 episodes, each with three runs.

The results of **Exp5** are summarized in Table 7. We observed high precision and recall for learning the missing axioms. Next, the results of **Exp6** are summarized in Figure 7; recall that this experiment had three consecutive runs in each of 10 episodes. By the end of the first run, the *ad hoc* agent successfully learned 4–5 of 14 missing objects, all three missing actions, 1–2 of three missing causal laws, and 6–7 of 10 missing executability conditions. After three consecutive runs, the number of discovered objects increased to 8–9 out of 14, 2–3 out of three causal laws, and 9–10 out of 10 executability conditions. The steady increase in number of objects, actions, and axioms learned, along with the high precision and recall, indicated the agent's ability to reliably learn new knowledge, thus supporting **H5**. Although the LLM occasionally provided incorrect ASP formats, these errors occurred rarely; most of the time the LLM output was correct as its use was limited to straightforward tasks and the mechanisms described in Section 3.5 were sufficient to account for these errors.

**Table 7:** Average precision and recall of learned axioms in 30 runs over 10 episodes; previously unknown axioms were learned accurately.

Axiom type	Precision	Recall
Causal laws	0.96	1.0
Executability conditions	1.0	1.0

Next, Table 8 summarizes the results of **Exp7**, which evaluated the impact of the learned knowledge on the ability to complete tasks as a team. We ran paired trials with and without the learned axioms and computed the value of these measures for the former (i.e., when the learned axioms are included) as a fraction of the values for the latter (i.e., without the learned axioms). Table 8 shows that when the *ad hoc* agent reasoned with the learned objects, actions, and axioms, it substantially improved performance compared with reasoning without this knowledge. In the absence of the learned knowledge, at least one *ad hoc* agent often could not compute valid plans to complete the tasks, and could not contribute to the team's performance. The team was essentially operating with one fewer member in such cases, with the other two members executing additional actions. The significant low number of steps and time (*p* < 0.0001) emphasize the importance of learning previously unknown knowledge, thus supporting **H6**.

**Table 8:** Average number of steps and time taken by the team to complete task sequences represented as a fraction of these values for the baseline; reasoning with learned knowledge substantially improved performance.

Architecture	Steps	Time(s)
With learned axioms	0.76	0.84
Without learned axioms	1.00	1.00

Lastly, Table 9 summarizes the results of **Exp8**. We observed high values of precision and recall indicating the ability of the agent to automatically extract the correct literals to provide relational descriptions as explanations in response to different types of questions. These results support **H7**.

**Table 9:** Precision and recall of retrieving relevant literals for providing explanations in the VirtualHome domain.

Question type	Precision	Recall
Action justification	1.00	1.00
Contrastive	0.89	0.99
Belief justification	0.88	0.94
Counterfactual	1.00	0.78

### 4.3 Qualitative results

**Figure 8:**
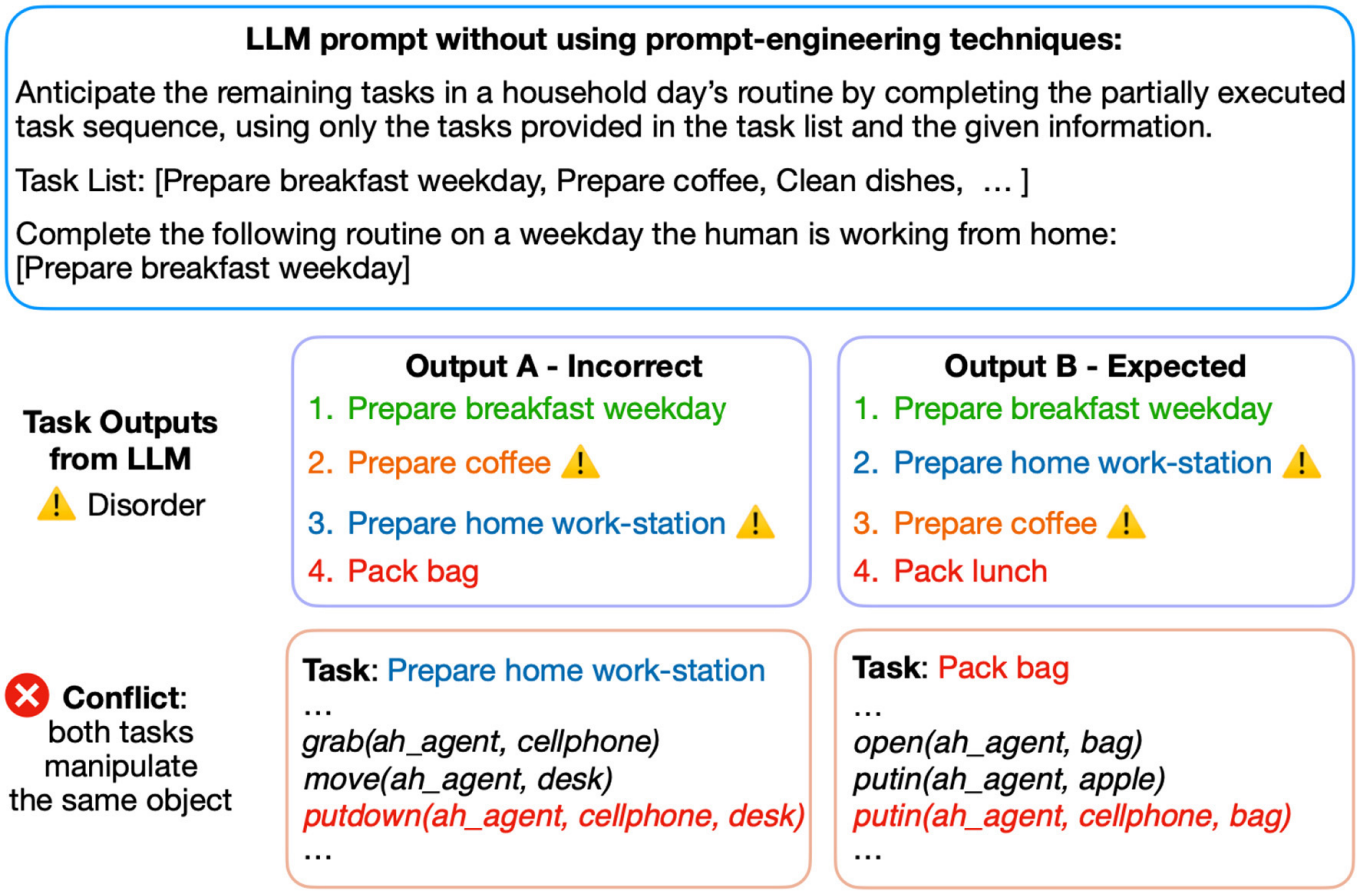
Execution example: using LLM without prompting strategies or external validator causes conflicts during execution, having a negative impact on performance.

We also provide some execution traces as a qualitative evaluation of **H3**. Figure 8 shows an execution example where the *ad hoc* agent used the LLM to anticipate future tasks, with and without the prompting strategies and validator. The example was set on a weekday where the human was working from home and no guests were expected. The correct task routine in this context was: *Prepare breakfast, Prepare home work-station, Prepare coffee, Prepare lunch*.

When the *ad hoc* agent queried the LLM without the prompt engineering techniques or validator (Section 3.3), the anticipated task list was different from the expected output. The prompt to the LLM without using the prompt engineering strategies is shown in Figure 8. The LLM output in this scenario was [*Prepare breakfast, Prepare coffee, Prepare home work-station, Pack bag*]. This output failed to align with the human preferences and priorities as:

- Higher priority was assigned to making coffee than setting up the workstation. This would delay the human for work and lead to coffee not being hot when needed.
- Packing the bag was an unnecessary task as the human was not leaving the house, and would have been filtered out by the validator.

These preferences and priorities were captured by the agent through its experience with the human across multiple iterations, and used by the validator to filter out irrelevant tasks or reorder them. When the *ad hoc* agent used the prompt engineering and validation strategies, the prompt to the LLM was automatically generated while incorporating context, as described in Section 3.3. The LLM's output was [*Prepare breakfast, Prepare home work-station, Prepare coffee, Prepare lunch*]. This matched the expected routine. i.e., making breakfast and setting up the workstation were considered high priority tasks, and irrelevant tasks such as *pack bag* were filtered out by the validator. These results demonstrate the importance of using a combination of prompting techniques and the external validator, supporting **H3**.

We observed similar situations when extending the setup to three agents, one human and two *ad hoc* agents, collaborating on a weekend when guests were expected. The correct task routine in this context was: *Prepare breakfast, Prepare table for guests, Prepare lunch, and Clean dishes*. When the first *ad hoc* agent queried the LLM with the prompting strategies but without the validator, the anticipated task list by the LLM was *Prepare breakfast, Prepare table for guests, Prepare lunch, and Serve snacks*. For the second *ad hoc* agent the LLM output was *Prepare breakfast, Prepare table for guests, Prepare lunch, Serve snacks, and Clean dishes*. When the validator was used, the outputs to both the agents were refined by incorporating context. Since the human usually did not require snacks after lunch, the task *Serve snacks* was removed. For the first agent the refined task list was *Prepare breakfast, Prepare table for guests, and Prepare lunch*. For the second agent the task list was *Prepare breakfast, Prepare table for guests, Prepare lunch, and Clean dishes*; since *Clean dishes* was a defined task in this domain, it was retained by the validator. This example further demonstrates the importance of using the validator and support **H3**.

**Figure 9:**
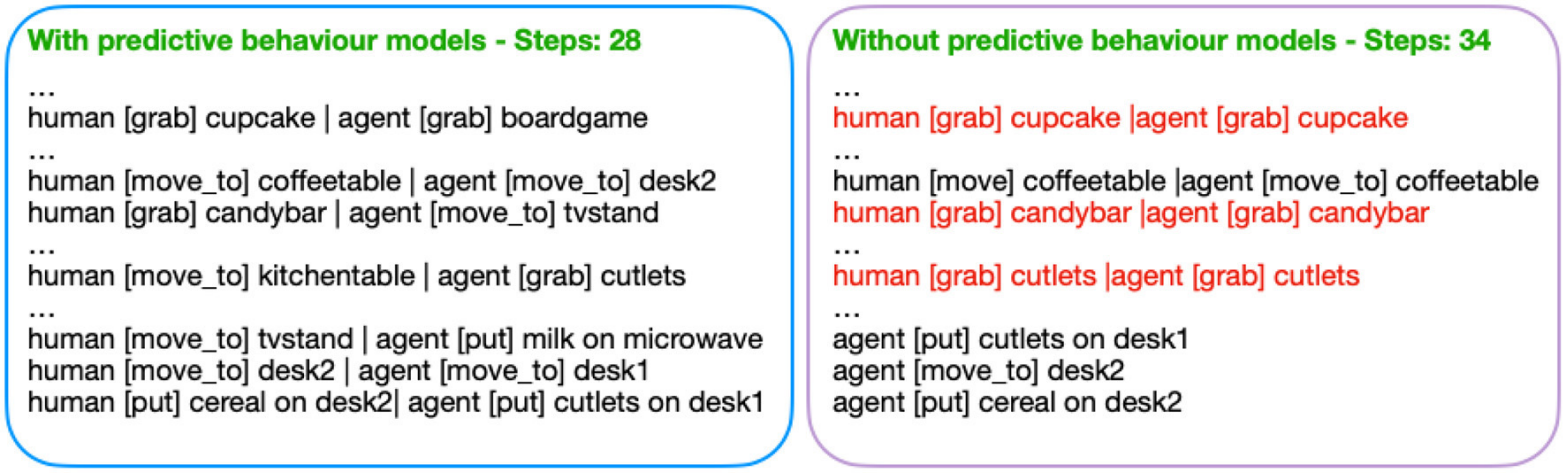
Execution trace for task routine: *[Prepare breakfast, Prepare activities, Serve snacks, Clean kitchen]*. When an *ad hoc* agent is not allowed to predict and reason about the human's actions, it may choose to execute same action(s) as the human, leading to longer plans.

Next, Figure 9 compares two plans executed by a team comprising a human and an *ad hoc* agent for completing a different set of tasks: *[Prepare breakfast, Prepare activities, Serve snacks, and Clean kitchen]*, with and without the behavior prediction models (Section 3.2). In the first plan, when the *ad hoc* agent used the behavior prediction model to predict the future actions of the human, the team successfully completed all tasks for the given day in just 28 steps. On the other hand, when the *ad hoc* agent did not use behavior prediction models, it often selected the same actions as the human for any particular task, leading to unnecessary delays in completing the tasks. For example, in the second plan the agent frequently selected the same action as the human—simultaneously picking up the cupcake, candy bar, and cutlets, introducing redundant behavior and prolonging task execution. As a result, the overall plan was extended to 34 steps. These results demonstrate that using the behavior prediction models enables the *ad hoc* agent to coordinate efficiently by avoiding action conflicts with the human. This further supports **H1**.

**Figure 10:**
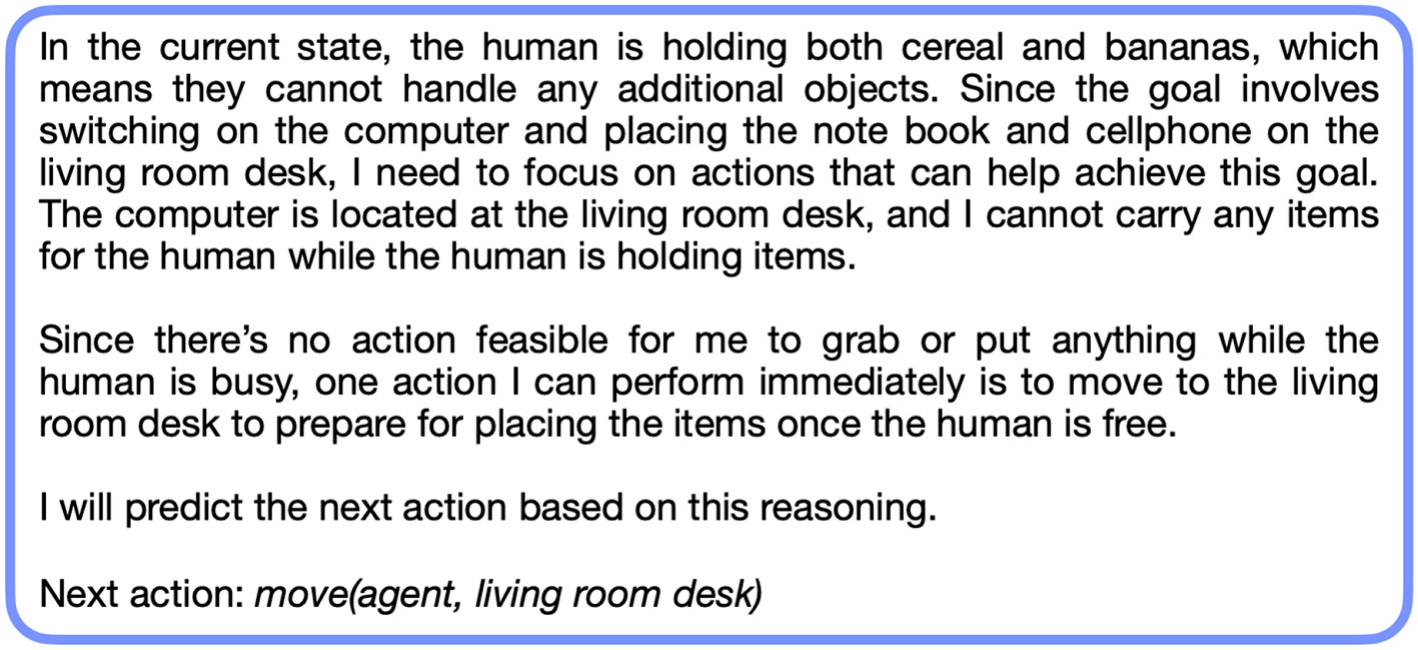
Example output when *ad hoc* agent uses the LLM for computing sequences of actions for specific tasks (**Base8**).

When the *ad hoc* agent used the LLM for directly computing sequences of actions for specific tasks **Base8**, the prompt was automatically constructed following the procedure described in Section 4.1. Figure 10 shows part of the LLM output during the task routine: [*Prepare breakfast, Prepare home work-station, Prepare coffee, and Prepare lunch*]. The selected action *move (agent, living room desk)* violated the “Movement Limitation (Critical)” rule in “Action Feasibility Rules,” which constrained the agent to only move to locations adjacent to its current location. This example demonstrates that the LLM may not respect constraints even when they are provided as input, and highlights that an LLM is not designed for computing plans for non-trivial tasks; using an LLM to directly output a sequence of low-level actions to complete tasks can lead to infeasible actions, resulting in poor performance. These results supports hypothesis **H4**.

Next, we provide some execution traces illustrating KAT's explanation generation capabilities. Consider the scenario in which the task is to *prepare breakfast* and the world state is: bread slice is inside the toaster; cutlets are on the kitchen counter; poundcake is on the kitchen table; water glass is in the bedroom; microwave is closed and switched off; frying pan is on the stove that is switched off; and the human and *ad hoc* agent are in the kitchen.

To help the human prepare breakfast, the *ad hoc* agent generated a plan with 23 steps, some of which are shown below; the agent expects the human to complete some intermediate steps.

**Table:** 

1 Find the bread slice	2 Pick up the bread slice from the toaster
3 Put the bread slice on the kitchen table	4 Find the cutlets
5 Pick up the cutlets from the kitchen counter	6 Find the stove
…	…
18 Put the poundcake inside the microwave	19 Close the microwave
20 Switch on the microwave	21 Find the cutlets
22 Pick up the cutlets from the frying pan	23 Put the cutlets on the kitchen table

**Execution example 1**. [Action Justification, Contrastive, Counterfactual] Consider an exchange with the *ad hoc* agent after executing first plan step.

- **Questioner:** “Why did you find bread slice in step 1?"
- ***ad hoc* Agent:** “Because I had not found the bread slice yet and I wanted to grab it in step 2".
  The agent's response draws attention to the target action's outcome being a requirement for executing a subsequent action. Specifically, since the *grab* action occurred immediately after *find*, the relevant axioms identified included:
$$\begin{array}{l} \neg occurs\left(grab\left(R,O\right),I\right)\leftarrow \neg holds\left(found\left(R,O\right),I\right), \end{array}$$
$$\begin{array}{l} agent\left(R\right),graspable\left(O\right). \end{array}$$ (9)
  Next, the *ad hoc* agent explored the relevant answer sets and identified that the literal *found(ad_hoc_agent, bread_slice)* was present in step 2 but not in step 1. This literal was then selected to construct the answer justifying the action execution. The agent can also be asked why it did not consider picking up a different object.
- **Questioner:** “Why did you not find the water glass in step 1?”
- ***ad hoc* Agent:** “Because I predicted that the human will find the water glass in step 1.”
  The agent first tried to identify the axioms with the action *find* in the head.
$$\begin{array}{l} \neg occurs\left(find\left(R,O\right),I\right)\leftarrow \neg holds\left(agent\text{\_}found\left(T,O\right),I\right), \end{array}$$
$$\begin{array}{l} other\text{\_}agents\left(T\right),graspable\left(O\right). \end{array}$$ (10)
  Next, the agent ground the body of the axiom and verified whether it was included in the answer set. Since the *agent_found(human, waterglass)* was valid in step 1, the agent identified it as a literal that prevented it from considering the *find* action in step 1. This is due to the *ad hoc* agent's model of the human predicting that the human will execute action *exo_find(human, waterglass)* in step 0. The agent may also be asked about the human's action choices further.
- **Questioner:** “Why do you think human will grab the water glass in step 2?”
- ***ad hoc* Agent:** “Because my prediction is that the human wants to bring the glass to the table.”
- **Questioner:** “What if the human decided to grab the cutlets in step 2?”
- ***ad hoc* Agent:** “If the human grabs the cutlets in step 2, they will be in the human's hands in step 3.”
  To answer questions about hypothetical situations, the *ad hoc* agent has to simulate the evolution of state, and the execution of actions by the human and the agent for a few steps in order to identify and report the motivation for specific action choices. Specifically, the agent first retrieved the current state estimate and initialized a new *VirtualHome* environment. Then it simulated the environment for the desired future time steps while using the human behavior model to predict the future actions of the human. Next, it went through the FF tree model predicting the human behavior and identified rules that caused it to believe the human will *grab* the water glass in step 2. this information was then used to generate the responses (above) to the questions posed.

**Execution example 2**. [Action Justification, Contrastive, Belief Tracing] The accuracy of the model predicting human action choices (85%) indicates that the predictions can be incorrect, particularly in situations in which the agent's understanding of the human is limited to a few observations. Consider a variant of the scenario above, in which the human decided to find and grab a slice of bread as the first action. Since the *ad hoc* agent decided to do the same action, it created a conflict. Some key steps of the plan computed by the *ad hoc* agent to overcome this conflict are shown below. The subsequent conversation between the questioner and the *ad hoc* agent is as follows:

**Table:** 

1 Find the cutlets	2 Pick up the cutlets from the kitchen counter
3 Find the frying pan	4 Put the cutlets inside the frying pan
5 Find stove	6 Switch on the stove
…	…
18 Close the microwave	19 Switch on the microwave
20 Switch off the microwave	21 Open the microwave
22 Pick up the poundcake from the microwave	23 Put the poundcake on the kitchen table

**Figure 11:**
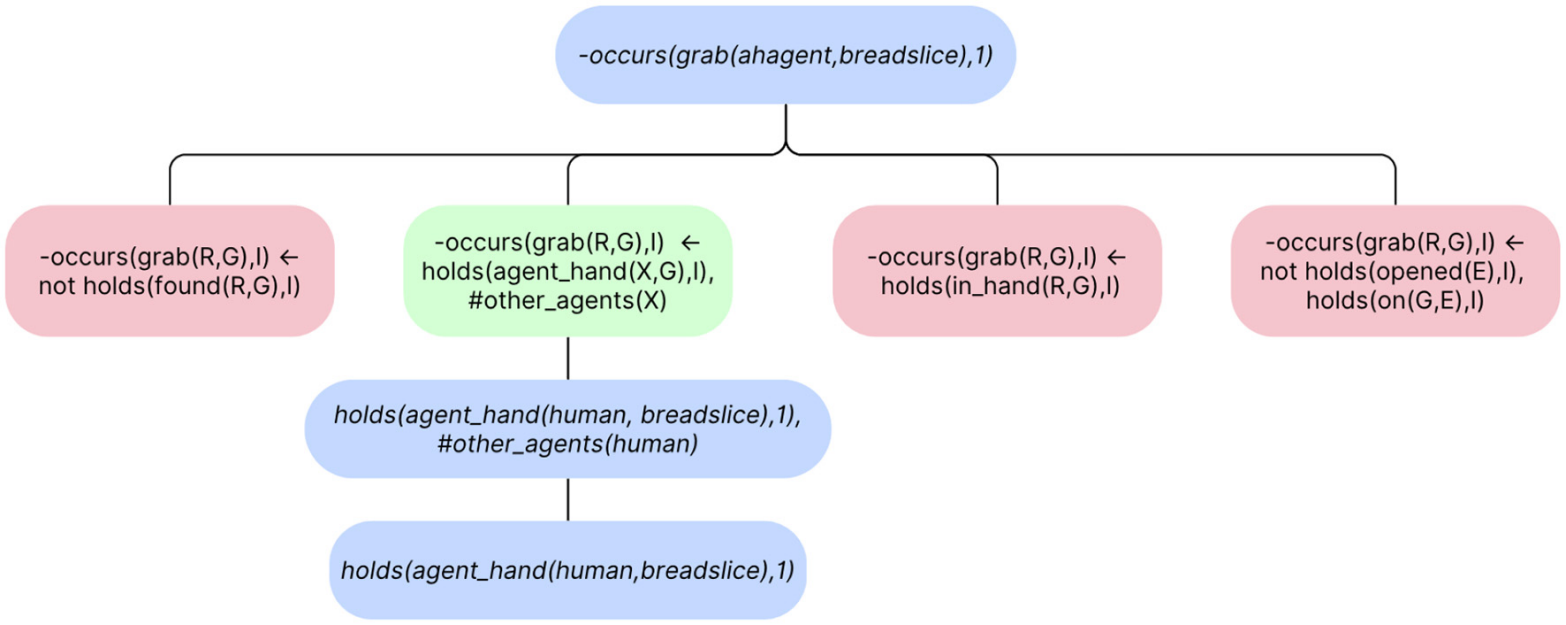
Belief tracing to explore why *ad hoc* agent did not grab bread slide in step 1.

- **Questioner:** “Why did you find cutlets in step 1?”
- ***ad hoc* Agent:** “Because I have not found the cutlets and I wanted to grab them in time step 2.”
- **Questioner:** “Why did you not grab bread slice in step 1?”
- ***ad hoc* Agent:** “Because the human was holding the bread slice.”
  This exchange demonstrates the ability of the *ad hoc* agent to change its plan in order to prevent a conflict with the human, and to justify this decision. Similar to the previous example, the *ad hoc* agent used the identified axioms and literals to generate the answer to the questions posed. Figure 11 shows the belief tree created for tracing the *ad hoc* agent's beliefs and justifying why it did not grab the bread slice in step 1. The green and blue boxes represent the satisfied axioms and their grounded literals, while the red boxes represent the axioms that were not satisfied in this example.

Overall, these results indicate the *ad hoc* agent's ability to generate relation descriptions as explanations of its decision and beliefs and those of the other agents and support hypothesis **H7**.

All source code for the architecture is available in our repository (Dodampegama and Sridharan, 2026).

## 5 Conclusions

This paper described KAT, an architecture that enables an AI agent to collaborate with other agents (human, AI) without prior coordination. KAT embeds the principles of refinement, ecological rationality, and interactive learning, enabling each *ad hoc* agent to: automatically identify and reason with relevant information; leverage the generic knowledge encoded in a pre-trained LLM for high-level task anticipation; rapidly learn models predicting the action choices of teammates; perform non-monotonic logical reasoning with prior knowledge and behavior models to plan and execute actions to jointly achieve the current and anticipated tasks; leverage a LLM to translate natural language descriptions of action outcomes into formal ASP representations of previously unknown objects, actions, and axioms; use decision tree induction to incrementally learn axioms based on observations; and generate on-demand explanations of the agent's decisions and beliefs and those of others as answers to different queries. Based on experiments in a realistic, physics-based simulation environment, we demonstrated performance improvements compared with various baselines, highlighting the significance of each component of our architecture and the ability to scale to additional agents. Future work will extend this approach to larger, heterogeneous teams and robots collaborating with humans in AHT settings.

## Data Availability

The datasets presented in this study can be found in online repositories. The names of the repository/repositories and accession number(s) can be found in the article/supplementary material.

## Footnotes

1 Communication is an important means to share information. However, since communication is not readily available in many practical AHT domains, we do not include it in our example scenario.
